# Identification of the Key Regulators of Spina Bifida Through Graph-Theoretical Approach

**DOI:** 10.3389/fgene.2021.597983

**Published:** 2021-04-06

**Authors:** Naaila Tamkeen, Suliman Yousef AlOmar, Saeed Awad M. Alqahtani, Abdullah Al-jurayyan, Anam Farooqui, Safia Tazyeen, Nadeem Ahmad, Romana Ishrat

**Affiliations:** ^1^Department of Biosciences, Jamia Millia Islamia, New Delhi, India; ^2^Centre for Interdisciplinary Research in Basic Sciences, Jamia Millia Islamia, New Delhi, India; ^3^Doping Research Chair, Department of Zoology, College of Science, King Saud University, Riyadh, Saudi Arabia; ^4^Department of Physiology, Faculty of Medicine, Taibah University, Medina, Saudi Arabia; ^5^Immunology and HLA Section, Pathology and Clinical Laboratory Medicine, King Fahad Medical City, Riyadh, Saudi Arabia

**Keywords:** Spina bifida, protein-protein interaction network, topological analysis, rich-club analysis, key regulators

## Abstract

Spina Bifida (SB) is a congenital spinal cord malformation. Efforts to discern the key regulators (KRs) of the SB protein-protein interaction (PPI) network are requisite for developing its successful interventions. The architecture of the SB network, constructed from 117 manually curated genes was found to self-organize into a scale-free fractal state having a weak hierarchical organization. We identified three modules/motifs consisting of ten KRs, namely, *TNIP1*, *TNF*, *TRAF1*, *TNRC6B*, *KMT2C*, *KMT2D*, *NCOA3*, *TRDMT1*, *DICER1*, and *HDAC1*. These KRs serve as the backbone of the network, they propagate signals through the different hierarchical levels of the network to conserve the network’s stability while maintaining low popularity in the network. We also observed that the SB network exhibits a rich-club organization, the formation of which is attributed to our key regulators also except for *TNIP1* and *TRDMT1*. The KRs that were found to ally with each other and emerge in the same motif, open up a new dimension of research of studying these KRs together. Owing to the multiple etiology and mechanisms of SB, a combination of several biomarkers is expected to have higher diagnostic accuracy for SB as compared to using a single biomarker. So, if all the KRs present in a single module/motif are targetted together, they can serve as biomarkers for the diagnosis of SB. Our study puts forward some novel SB-related genes that need further experimental validation to be considered as reliable future biomarkers and therapeutic targets.

## Introduction

The vigorous efforts of researchers and an increase in research techniques have paved the way for discoveries in the study of polygenic and other complex human diseases. However, there remain a large fraction of genetic diseases with their molecular basis unknown, Spina bifida (SB) being one of them. SB, a spinal cord malformation, falls into the category of Neural Tube Defects (NTDs). This birth defect occurs when the neural tube of an embryo fails to close completely during the 4th week of pregnancy. As a result, the vertebral column remains split (bifid). Myelomeningocele (MMC) is the most common and serious type of SB and is often used interchangeably with it. MMC causes partial/complete loss of all the functions below the level of injury. Defective bladder and bowel functions, hindbrain herniation (Chiari II malformation), hydrocephalus, orthopedic abnormalities also exist in these patients (Copp et al., 2015; Mohd-Zin et al., 2017). Apart from MMC, other open and closed forms of SB are also there (Brei and Houtrow, 2017).

Etiologically, SB involves both genetic as well as environmental factors. Though the genetic component of SB is believed to be at 60–70%, yet, non-genetic risk factors like reduced folate intake, maternal anticonvulsant therapy, diabetes mellitus, and obesity, cannot be ruled out on account of some important studies, for example, the one which states that up to 70% of NTDs in the general population can be prevented by periconceptional maternal supplementation with folic acid. Although SB has received much attention over the last decades because of its high prevalence of one case per 1,000 births globally, its exact causation is yet to be deciphered. The mechanism by which folic acid protects against SB also remains a challenge for the researchers despite its unambiguous impact on the disorder (Beaudin and Stover, 2007; Copp et al., 2015). Individuals with SB need medical management throughout their lives apart from surgical treatment (Mitchell et al., 2004).

A disease is more often a consequence of the perturbations of the complex intracellular network of interactions between functionally related genes, rather than a single gene abnormality. This led to the systemic approach to biological problems which is based on the principle that to understand the contribution of various genes/proteins in disease initiation and progression, one has to look at the network of interactions of a living system as a whole. Here comes into picture the concept of Network medicine, which aims to explore the complexity of a disease through the systematic identification of the disease pathways and modules. Here, the protein interaction maps are developed and later analyzed through graph/network theory to understand the theoretical aspect of complex networks (Re et al., 2008; Barabási et al., 2011). According to the graph theory, analysis of the topological structure of a network provides important information of the network through which novel disease genes and pathways, biomarkers, and drug targets for complex diseases can be identified (Browne et al., 2018; Chen et al., 2019).

A recent study on the complex protein-protein interaction (PPI) network suggests its conformity to scale-free topology on a hierarchical scale. On these networks, the problem arises that the central lethality rule does not apply where the stability and dynamics of the network are disrupted but not completely disrupted when the hubs are targeted (Ali et al., 2018). This may be due to the hierarchical organization of modules/sub-modules in complex networks and other biological networks at various topological levels, where specific roles are associated with them (Farooqui et al., 2018; Malik et al., 2019; Mangangcha et al., 2019, 2020). We followed similar methods while conducting this study.

Thus, the focus of our study is on the Protein-Protein Interaction (PPI) network/graph of SB, constructed from manually curated genes with an aim to understand the topology or the architectural principle of the network/graph (random, small world, scale-free or hierarchical) which is a prerequisite to identify the main key drivers/regulators of the network. We also analyzed a rich-club structural ordering of the SB network that signifies an efficient higher-order organization indicating the existence of the rich-club nodes which increase the network’s stability as a whole. We further extended our study to the identification of the modules/motifs consisting of the important key regulators of the network that have fundamental importance due to their activities and regulating mechanisms in the network based on previous successful applications of related methodologies.

## Materials and Methods

### Data Mining and Manual Curation of the Candidate Genes Involved in Spina Bifida (SB)

The candidate genes of human SB were manually curated from literature through databases i.e., gene, PubMed, and OMIM (Hamosh et al., 2005), housed in NCBI. We got a list of 117 experimentally verified genes that we used as seed genes in the construction of the SB network.

### Construction of Protein-Protein Interaction Network of SB

The candidate genes were mapped to their respective proteins to construct the Protein-Protein Interaction (PPI) network of SB in the STRING database (The Search Tool for the Retrieval of Interacting Genes^1^) (Szklarczyk et al., 2017) with an interaction score >0.50 as the threshold, following the concept of one gene-one protein. To construct the primary PPI network from the seed genes, we added 500 interactors in the first shell and 500 interactors in the second shell. Subsequently, the network was visualized and analyzed in the Cytoscape software (version 3.6.1) (Shannon et al., 2003).

### Topological Properties Analysis of the SB Network

Networks have some properties, called topological properties which serve to unravel the information that they contain. The topological properties which were studied to delve into the important behavior of the constructed primary SB PPI network are degree distribution, neighborhood connectivity, clustering coefficient, betweenness centrality, closeness centrality, and eigenvector centrality. All of these were calculated using Network Analyzer, a plug-in in Cytoscape version 3.6.1, except for eigenvector centrality, which was calculated using CytoNCA (Tang et al., 2015), another plug-in in Cytoscape. The topological properties analysis was required to validate that the SB network is following the criteria of a scale-free and hierarchical network because in biological systems, networks are arranged in a scale-free manner. Al Barabasi et al. (Barabási and Albert, 1999) proposed a model based on the mechanisms that most real networks expand continuously by the addition of new vertices and the new vertices attach preferentially to already well-connected sites. This led to the observation that many large networks have a common property that their vertex connectivities follow a scale-free power-law distribution, meaning that in large complex networks the probability that a vertex in the network interacts with k other vertices decays as a power law, following *P(k) ∼ k^–γ^*. The fitting parameter values are given by r^2 = 0.410. This result indicates that large networks self-organize into a scale-free state despite their continuous growth.

#### Degree Distribution

The degree *k* of a node is a local measure of centrality of that node, it is the number of edges by which that node is linked to other nodes in a network (Raman, 2010). Degree distribution *P*(*k*), is the distribution of the node degrees over the whole network. *P*(*k*) gives the probability that a randomly selected node has exactly *k* links and is calculated by dividing the number of nodes (*n*_*k*_) having a degree *k* (with *k* = 1,2,3…) by the total number of nodes (*N*) in the network;

(1) $$P\left(k\right)=\frac{n_{k}}{N}$$

*P*(*k*) decays as a power-law *P*(*k*)∼*k*^–γ^ for a scale-free network, where, *γ* is the degree exponent, whereas, the value of *γ* becomes close to *γ* ∼ 2.26 (mean-field value) for hierarchical networks and if a network is random then *P*(*k*) follows poisson distribution, thus, *P*(*k*) can be used to distinguish between various network topologies (Albert and Barabási, 2002).

#### Neighborhood Connectivity

The set of neighbors of a given node *n* is the node’s neighborhood and the number of its neighbors is its connectivity. The neighborhood connectivity of the node *n* is defined as the average connectivity of all the nearest neighbors of *n* (Maslov and Sneppen, 2002). Neighborhood connectivity is given by,

(2) $$C_{N}\left(k\right)=\Sigma_{q}qP\left(\frac{q}{k}\right)$$

where, *P* $\left(\frac{q}{k}\right)$ is the conditional probability that a link belonging to a node with connectivity *k* points to a node with connectivity *q*. While *C*_*N*_(*k*) obeys power law in the case of a hierarchical network, *C*_*N*_(*k*) ∼ *k*^–β^ with β ∼ 0.5, for a scale-free network, *C*_*N*_(*k*) ∼ constant (Pastor-Satorras et al., 2001; Malik et al., 2017). Positive and negative power dependence of *C*_*N*_(*k*) could be the indicators of assortativity and disassortativity in the network topology, respectively (Barrat et al., 2004), meaning that if *C*_*N*_(*k*) follows power-law with a positive value of exponent β (i.e., *C*_*N*_(*k*) ∼ *k*^+β^) then edges between highly connected nodes prevail in a network, this shows assortative nature of the network, whereas, if *C*_*N*_(*k*) follows power-law with a negative value of exponent β (i.e., *C*_*N*_(*k*) ∼ *k*^–β^) then edges between lowly connected and highly connected nodes prevail in a network, this shows disassortative nature of the network.

#### Clustering Coefficient

The clustering coefficient, for a node *n*, is a notion of how connected the neighbors of that node are, in a network. The clustering coefficient for *n* is a ratio of the number of edges between the neighbors of *n*, and the maximum number of edges that could possibly exist between the neighbors of *n*. For an undirected network, clustering coefficient *C*(*k*_*n*_) of a node *n* can be calculated by,

(3) $$C\left(k_{n}\right)=\frac{2e_{n}}{k_{n}\left(k_{n}-1\right)}$$

where, *e*_*n*_ is the number of connected pairs between all nearest-neighbors of the node *n*, and *k*_*n*_ is the degree of the node *n* (Ravasz and Barabási, 2003).

The clustering coefficient, when applied to an entire network is called the average clustering coefficient and is defined as the average of the clustering coefficients for all nodes in that network, it gives a measure of the tendency of the nodes in that network to cluster together.

#### Betweenness Centrality

Betweenness centrality is a measure of the frequency of occurrence of a node on all shortest paths between all pairs of nodes in a network (Brandes, 2001). The betweenness centrality of a node indicates the amount of influence it has over the flow of information in a network by behaving like an interaction bridge between other nodes in the network. The betweenness centrality *C_*B*_(n)* of a node *n* is given by the expression:

(4) $$C_{B}\left(n\right)=\sum_{s\neq n\neq t}\left(\frac{\sigma_{st}\left(n\right)}{\sigma_{st}}\right)$$

where, *s* and *t* are nodes in the network other than *n*, *σ_*st*_* is the total number of shortest paths from *s* to *t*, and *σ_*st*_* (*n*) is the number of those shortest paths from *s* to *t* on which *n* lies (Albert and Barabási, 2002; Maslov and Sneppen, 2002; Raman, 2010).

#### Closeness Centrality

Closeness centrality (*C*_*C*_) is a measure of how close a node is, to all the other nodes reachable from it in a network, thus, it points out the nodes which are able to spread information very efficiently through a network (Canright and Engø-Monsen, 2004). *C*_*C*_(*n*) of a node *n* is defined as the reciprocal of the average length of the shortest paths between *n* and all other nodes connected to it in the network, and is given by,

(5) $$C_{C}\left(n\right)=\frac{N-1}{\sum_{j}d_{ij}}$$

where, *dij* is the length of the shortest path between two nodes *i* and *j*, and *N* is the total number of nodes in the network which are connected to the node *n*.

#### Eigenvector Centrality

Eigenvector centrality is a measure of a node’s power to facilitate the spread of information in a network. Eigenvector centrality *C*_*E*_(*n*) of a node *n* in a network is proportional to the sum of it’s nearest neighbors centralities, and is defined by the equation,

(6) $$C_{E}\left(n\right)=\frac{1}{\lambda }\sum j=nn\left(n\right)v_{j}$$

where, *nn*(*n*) indicates the nearest neighbors of node *n* in the network. In the eigenvalue equation, *Av_*n*_* = *λv_*n*_*, *λ* is the eigenvalue of the eigenvector *v*_*i*_ and *A* is the adjacency matrix (connection matrix) of the network. The principal eigenvector of *A*, which corresponds to maximum eigenvalue *λ_*max*_*, is considered to have a positive eigenvector centrality score (Barrat et al., 2004; Malik et al., 2017).

### Community/Modules Detection

Characterization of the modular framework of the constructed network was requisite to define its behavior as a hierarchical network having several levels of hierarchy (Traag et al., 2011, 2013). To detect communities that are distributed in a hierarchical fashion within the constructed network, we followed Newman and Girvan’s community finding algorithm (Newman and Girvan, 2004) and the method used was Leading Eigen Vector method (LEV) (Newman, 2006) in R using ‘‘igraph’’ package^2^, LEV computation is considered fairly reliable since it reckons the eigenvalue of each edge, thus, indicating the significance of edges rather than nodes. We kept breaking the network into modules and then sub-modules till the motif level came which is the last level of network organization after which the network can not be broken further. Identifying any sub-module as a community was based on the criterion that it should be found to contain at least one triangular motif [defined by G(3, 3) i.e., 3 nodes and 3 edges].

Modularity is used to estimate the strength of the division of a network into communities (Newman and Girvan, 2004). If *m* denotes the total number of edges in a community, *Aij* is the adjacency matrix of size *i* × *j*, *k* represents degrees, and the *δ* function yields 1 if nodes *i* and *j* are in the same community, then Modularity (Q) of the community can be defined as,

(7) $$Q=\frac{1}{2m}\sum_{ij}\left(A_{ij}-\frac{k_{i}k_{j}}{2m}\right)\delta \left(C_{i},C_{j}\right)$$

### Functional Enrichment and Pathway Enrichment of the SB Network’s Seed Genes

In the current study, we used The Database for Annotation, Visualization and Integrated Discovery (DAVID) which extracts biological meaning from large lists of genes (Newman, 2005; Mason and Verwoerd, 2007) for the functional enrichment and pathway enrichment analysis of the seed genes (Huang et al., 2009a, b). The functions and pathways with adjusted Benjamini-adjusted *P*-value < 0.01 were considered statistically significant.

### Applying Local-Community-Paradigm (LCP) Approach to Estimate the Network’s Compactness

The LCP theory can be used to measure the self-organizing ability of a network by means of providing information regarding the number, size, and compactness of communities in the network. Thus, to check for the self-organizing ability of the SB network at each level of the organization, the LCP technique was employed using MATLAB 8.2.

A Local Community (LC) is composed of a cohort of Common Neighbors (CNs) of a given link and their cross-interactions-Local Community Links (LCLs) (Cacciola et al., 2019). The CN index between two nodes x and y, is the amount of overlap between their sets of first-node-neighbors S(x) and S(y) given by, CN = (Sx)∩S(y), a large value of CN indicates that these two nodes are more likely to interact, therefore, increase in CN reflects an increase in compactness in the network, which eventually indicates faster information processing in the network. The LCLs between x and y, whose upper bound is defined by, max(LCL) = 1/2CN(CN-1), are the number of internal links in the local-community (LC) (Cannistraci et al., 2013).

According to the LCP theory, the number of CNs of each link in a complex network is positively correlated with the respective number of LCLs, this led to a new network measure called local-community-paradigm correlation (LCP-corr). High LCP-corr values (usually >0.8) suggest rapid delivery of information across the various network modules and local processing by the formation of new links between CNs, thus, suggesting more dynamic self-reorganization in a network. On the other hand, low LCP-corr values (usually <0.4) characterize energetically expensive connections, thereby, indicating weak interactions between the nodes in non-LCP networks (Cacciola et al., 2019). The LCP correlation (LCP-corr) is the Pearson correlation coefficient between the variables CN and LCL and the formula for computing it is:

(8) $$LCP_{corr}=\frac{cov\left(CN,LCL\right)}{\sigma_{CN}.\sigma_{LCL}}$$

with CN > 1, where cov(CN, LCL) is the covariance between CN and LCL, σ_*CN*_ and σ_*LCL*_ are standard deviations of CN and LCL, respectively.

### Tracking of the Seed Genes Through the Networks

The path that each of the seed genes followed in the SB network, was tracked through various modules and sub-modules of the network and the ones which reached the motif level, as well as their interacting partners, were identified as the Key Regulators (KRs) of the network. In graph theory, KRs are considered significant as they serve as the backbone of a network from top to bottom organization and vice versa to maintain the network’s stability.

### Knock-Out Experiment of the Motifs Consisting of the Key Regulators

The deletion of the KRs might result in lethality on account of their regulatory role in a network. The regulation exerted by the KRs in a network can be understood theoretically, by studying the changes observed in the connections and topological parameters of that network after the KRs being knocked-out from it, compared to the corresponding unmodified network. In this regard, the motifs consisting of the KRs that were identified in the last level of the network organization were separately removed from the constructed primary network, and the topological properties of the modified network were again calculated to discern the perturbations led by the knock-out of these motifs from the network. We repeated the *in silico* knockout experiment of the motifs successively at each level of the network systematization to comprehend the regulatory role of the constituent KRs in the network.

### Distribution of Energy in the Network: Hamiltonian Energy Calculation

At each level of the organization of a network, be it a global or a modular level, a certain level of energy is required to keep the network organized at that level. This can be measured by calculating Hamiltonian Energy (HE) of the network at that level within the formalism of the Constant Potts Model (Newman and Girvan, 2004; Canright and Engø-Monsen, 2006). HE of a network/module/sub-module can be calculated by,

(9) $$H^{\left[c\right]}=-\sum_{c}\left[e_{c}-\gamma n_{c}^{2}\right]$$

where, *c* is any community containing *e*_*c*_ and *n*_*c*_ number of edges and nodes, respectively, and *γ* is the resolution parameter acting as edge density threshold which is set to be 0.5. To demonstrate the regulation exerted by the KRs in the same combination as they were found in the motifs, HE calculation and comparison of the original and the motifs knockout networks were done (Malik et al., 2019).

### Rich-Club Analysis

The “rich-club” organization is attributed to the preferable linking of high-degree nodes (rich nodes) among themselves to form tight and well-interlinked sub-graphs (clubs) in a network (Colizza et al., 2006). The rich-club organization of a network can be discerned by computing the rich-club coefficient ϕ(*k*) across the degree range. If *N* > *k* denotes the number of nodes having a degree higher than a given value *k* and *E* > *k* stands for the number of links connecting the *N* > *k* nodes then ϕ(*k*) can be defined as:

(10) $$\phi \left(k\right)=\frac{2E_{>k}}{N_{>k}\left(N_{>k}-1\right)}$$

The rich-club coefficient ϕ(*k*) measures the connectedness of the rich nodes, by giving the ratio of the actual to the maximum number of links possible between the *N* > *k* nodes. However, a monotonic increase of ϕ(*k*) can often be misleading as it may rather be a consequence of a higher probability of high-degree nodes to share edges as compared to low-degree nodes. Therefore, ϕ(*k*) should be normalized by comparing it with those obtained from the maximally random networks with similar size and degree distribution in order to precisely assess the rich-club structural ordering. The normalized rich-club coefficient ϕ_*norm*_(*k*) is computed as:

(11) $$\phi_{norm}\left(k\right)=\frac{\phi \left(k\right)}{\phi_{rand}\left(k\right)}$$

Where, ϕ_*rand*_(*k*) is the average rich-club coefficient of the maximally random networks (Nunes Amaral and Guimera, 2006). ϕ_*norm*_(*k*) > 1 is the signature of a rich-club organization in a network. In contrast, ϕ_*norm*_(*k*) < 1 signifies a lack of interconnectivity among the rich nodes. We evaluated the existence of a rich-club structural ordering in the SB network which is known to aid in increasing a network’s stability (Mangangcha et al., 2019, 2020).

All the graphs were drawn using OriginPro 8.5 and the figures using Adobe Illustrator CS6.

## Results

### Data Acquisition Through Manual Curation

A list of 117 genes that were experimentally verified to be involved in Spina Bifida (SB) in humans was retrieved through manual curation of literature (Table 1). These genes were used for the construction of the SB network to understand the molecular mechanism behind the occurrence of the malformation and to provide a new angle for its future biomarkers and therapeutic targets.

**TABLE 1:** List of genes reported as risk factors for SB in humans.

S. no.	Gene Name	Gene ID	Description	Location	References
1	*ALDH1A2*	8854	Aldehyde dehydrogenase 1 family member A2	15q21.3	Deak et al., 2005b
2	*ALDH1L1*	10840	Aldehyde dehydrogenase 1 family member L1	3q21.3	Franke et al., 2009
3	*AMBRA1*	55626	Autophagy and beclin 1 regulator 1	11p11.2	Ye et al., 2020
4	*APAF1*	317	Apoptotic peptidase activating factor 1	12q23.1	Spellicy et al., 2018
5	*APEX1*	328	Apurinic/apyrimidinic endodeoxyribonuclease 1	14q11.2	Olshan et al., 2005
6	*APOB*	338	Apolipoprotein B	2p24.1	Zou et al., 2020
7	*BHMT*	635	Betaine–homocysteine S-methyltransferase	5q14.1	Shaw et al., 2009
8	*BMP4*	652	Bone morphogenetic protein 4	14q22.2	Felder et al., 2002
9	*BRCA1*	672	BRCA1, DNA repair associated	17q21.31	King et al., 2007
10	*CARM1*	10498	Coactivator associated arginine methyltransferase 1	19p13.2	Lu et al., 2010
11	*CASP8*	841	Caspase 8	2q33.1	Wang et al., 2017
12	*CBSL*	875	Cystathionine beta-synthase	21q22.3	Martinez et al., 2009
13	*CCL2*	6347	C-C motif chemokine ligand 2	17q12	Jensen et al., 2006a; Lu et al., 2008
14	*CDH2*	1000	Cadherin 2	18q12.1	Hebert et al., 2020
15	*CELSR1*	9620	Cadherin EGF LAG seven-pass G-type receptor 1	22q13.31	Lei et al., 2014
16	*CFL1*	1072	Cofilin 1	11q13.1	Zhu et al., 2007
17	*CHKA*	1119	Choline kinase alpha	11q13.2	Enaw et al., 2006
18	*CITED2*	10370	Cbp/p300 interacting transactivator with Glu/Asp rich carboxy-terminal domain 2	6q24.1	Lu et al., 2010
19	*COMT*	1312	Catechol-O-methyltransferase	22q11.21	Carter et al., 2011
20	*CPE*	1363	Carboxypeptidase E	4q32.3	Hebert et al., 2020
21	*CREBBP*	1387	CREB binding protein	16p13.3	Lu et al., 2010
22	*CSNK1G2*	1455	Casein kinase 1 gamma 2	19p13.3	Hebert et al., 2020
23	*CUBN*	8029	Cubilin	10p13	Franke et al., 2009
24	*CYP26A1*	1592	Cytochrome P450 family 26 subfamily A member 1	10q23.33	Rat et al., 2006
25	*DACT1*	51339	Disheveled binding antagonist of beta catenin 1	14q23.1	Shi et al., 2012
26	*DDB1*	1642	Damage specific DNA binding protein 1	11q12.2	Hebert et al., 2020
27	*DHFR*	1719	Dihydrofolate reductase	5q14.1	Martinez et al., 2009
28	*DLC1*	10395	DLC1 Rho GTPase activating protein	8p22	Le et al., 2018
29	*DVL1*	1855	Disheveled segment polarity protein 1	1p36.33	Chen et al., 2017
30	*DVL2*	1856	Disheveled segment polarity protein 2	17p13.1	De Marco et al., 2013
31	*EP300*	2033	E1A binding protein p300	22q13.2	Lu et al., 2010
32	*ERCC2*	2068	Xeroderma pigmentosum D	19q13.32	Olshan et al., 2005
33	*FERMT2*	10979	Fermitin family member 2	14q22.1	Hebert et al., 2020
34	*FKBP8*	23770	FKBP prolyl isomerase 8	19p13.11	Tian et al., 2020
35	*FOLH1*	2346	Folate hydrolase 1	11p11.12	Guo et al., 2013
36	*FOLR1*	2348	Folate receptor alpha	11q13.4	Findley et al., 2017
37	*FOLR2*	2350	Folate receptor beta	11q13.4	O’Byrne et al., 2010
38	*FOLR3*	2352	Folate receptor gamma	11q13.4	O’Byrne et al., 2010
39	*FOXN1*	8456	Forkhead box N1	17q11.2	Amorosi et al., 2008
40	*FUZ*	80199	Fuzzy planar cell polarity protein	19q13.33	Seo et al., 2011
41	*FZD3*	7976	Frizzled class receptor 3	8p21.1	Shangguan et al., 2015
42	*FZD6*	8323	Frizzled class receptor 6	8q22.3	De Marco et al., 2011; Juriloff and Harris, 2012
43	*GCKR*	2646	Glucokinase regulator	2p23.3	Fu et al., 2015
44	*GLI2*	2736	GLI family zinc finger 2	2q14.2	Lu et al., 2016
45	*GNAS*	2778	GNAS complex locus	20q13.32	Zhang et al., 2015
46	*GPC5*	2262	Glypican 5	13q31.3	Bassuk et al., 2013
47	*GPR161*	23432	G protein-coupled receptor 161	1q24.2	Kim et al., 2019
48	*GRHL3*	57822	Grainyhead like transcription factor 3	1p36.11	Le et al., 2017
49	*HK1*	3098	Hexokinase 1	10q22.1	Davidson et al., 2008
50	*HOXB7*	3217	Homeobox B7	17q21.32	Rochtus et al., 2015
51	*ITGB1*	3688	Integrin subunit beta 1	10p11.22	Le et al., 2018
52	*ITPK1*	3705	Inositol-tetrakisphosphate 1-kinase	14q32.12	Guan et al., 2014
53	*LEP*	3952	Leptin	7q32.1	Lupo et al., 2012
54	*LEPR*	3953	Leptin receptor	1p31.3	Carter et al., 2011; Suazo et al., 2013
55	*LMNB1*	4001	Lamin B1	5q23.2	Robinson et al., 2013
56	*LRP6*	4040	LDL receptor related protein 6	12p13.2	Lei et al., 2015
57	*MTHFD1*	4522	Methylenetetrahydrofolate dehydrogenase, cyclohydrolase and formyltetrahydrofolate synthetase 1	14q23.3	Parle-McDermott et al., 2006
58	*MTHFD1L*	25902	Methylenetetrahydrofolate dehydrogenase (NADP+ dependent) 1 like	6q25.1	Parle-McDermott et al., 2009
59	*MTHFD2*	10797	Methylenetetrahydrofolate dehydrogenase (NADP+ dependent) 2	2p13.1	Shaw et al., 2009
60	*MTHFR*	4524	Methylenetetrahydrofolate reductase	1p36.22	Martinez et al., 2009; Carter et al., 2011; Cadenas-Benitez et al., 2014
61	*MTR*	4548	5-methyltetrahydrofolate-homocysteine methyltransferase	1q43	Shaw et al., 2009
62	*MTRR*	4552	5-methyltetrahydrofolate-homocysteine methyltransferase reductase	5p15.31	van der Linden et al., 2006
63	*NAT1*	9	N-acetyltransferase 1	8p22	Jensen et al., 2006b
64	*NCAM1*	4684	Neural cell adhesion molecule 1	11q23.2	Deak et al., 2005a
65	*NKX2-8*	26257	NK2 homeobox 8	14q13.3	Safra et al., 2013
66	*NOG*	9241	Noggin	17q22	Felder et al., 2002
67	*NOS3*	4846	Nitric oxide synthase 3	7q36.1	Brown et al., 2004
68	*PAX1*	5075	Paired box 1	20p11.22	Hol et al., 1996
69	*PAX3*	5077	Paired box 3	2q36.1	Agopian et al., 2013
70	*PCMT1*	5110	Protein-L-isoaspartate (D-aspartate) O-methyltransferase	6q25.1	Zhu et al., 2006
71	*PCYT1A*	5130	Phosphate cytidylyltransferase 1, choline, alpha	3q29	Enaw et al., 2006
72	*PDGFRA*	5156	Platelet derived growth factor receptor alpha	4q12	Zhu et al., 2004; Carter et al., 2011
73	*PLCB1*	23236	Phospholipase C beta 1	20p12.3	Hebert et al., 2020
74	*PON1*	5444	Paraoxonase 1	7q21.3	Gonzalez-Herrera et al., 2010
75	*PORCN*	64840	Porcupine O-acyltransferase	Xp11.23	Hebert et al., 2020
76	*PPP2R1A*	5518	Protein phosphatase 2 scaffold subunit alpha	19q13.41	Hebert et al., 2020
77	*PRICKLE1*	144165	Prickle planar cell polarity protein 1	12q12	Bosoi et al., 2011
78	*PRICKLE2*	166336	Prickle planar cell polarity protein 2	3p14.1	Hebert et al., 2020
79	*PSMD3*	5709	Proteasome 26S subunit, non-ATPase 3	17q21.1	Hebert et al., 2020
80	*PTCH1*	5727	Patched 1	9q22.32	Wang et al., 2013b
81	*PTK7*	5754	Protein tyrosine kinase 7	6p21.1	Lei et al., 2019
82	*PTPRS*	5802	Protein tyrosine phosphatase receptor type S	19p13.3	Le et al., 2015
83	*PTPRU*	10076	Protein tyrosine phosphatase receptor type U	1p35.3	Hebert et al., 2020
84	*RAD9B*	144715	RAD9 checkpoint clamp component B	12q24.11	Cao et al., 2020
85	*RFC1*	5981	Replication factor C subunit 1	4p14	De Marco et al., 2003
86	*SARDH*	1757	Sarcosine dehydrogenase	9q34.2	Franke et al., 2009
87	*SCRIB*	23513	Scribbled planar cell polarity protein	8q24.3	Lei et al., 2013
88	*SDC1*	6382	Syndecan 1	2p24.1	Hebert et al., 2020
89	*SEC24B*	10427	SEC24 homolog B, COPII coat complex component	4q25	Yang et al., 2013
90	*SHMT1*	6470	Serine hydroxymethyltransferase 1	17p11.2	Rebekah et al., 2017
91	*SHROOM2*	357	Shroom family member 2	Xp22.2	Chen et al., 2018
92	*SHROOM3*	57619	Shroom family member 3	4q21.1	Le et al., 2015
93	*SLC19A1*	6573	Solute carrier family 19 member 1	21q22.3	O’Byrne et al., 2010
94	*SLC2A1*	6513	Solute carrier family 2 member 1	1p34.2	Davidson et al., 2008; Suazo et al., 2013
95	*SMO*	6608	Smoothened, frizzled class receptor	7q32.1	Wang et al., 2013a
96	*SOD1*	6647	Superoxide dismutase 1	21q22.11	Kase et al., 2012; Kase et al., 2013
97	*SOD2*	6648	Superoxide dismutase 2	6q25.3	Kase et al., 2012; Kase et al., 2013
98	*SOSTDC1*	25928	Sclerostin domain containing 1	7p21.2	Hebert et al., 2020
99	*SOX18*	54345	SRY-box transcription factor 18	20q13.33	Rochtus et al., 2016
100	*SOX3*	6658	SRY-box transcription factor 3	Xq27.1	Bauters et al., 2014
101	*T*	6862	T-box transcription factor T	6q27	Carter et al., 2011
102	*TFAP2A*	7020	Transcription factor AP-2 alpha	6p24.3	Lu et al., 2010
103	*TLE3*	7090	TLE family member 3, transcriptional corepressor	15q23	Hebert et al., 2020
104	*TNIP1*	10318	TNFAIP3 interacting protein 1	5q33.1	Francesca et al., 2016
105	*TNRC6B*	23112	Trinucleotide repeat containing adaptor 6B	22q13.1	Hebert et al., 2020
106	*TP53*	7157	Tumor protein p53	17p13.1	Pangilinan et al., 2008
107	*TRDMT1*	1787	tRNA aspartic acid methyltransferase 1	10p13	Franke et al., 2009
108	*TRIM26*	7726	Tripartite motif containing 26	6p22.1	Zhang et al., 2015
109	*TRIM4*	89122	Tripartite motif containing 4	7q22.1	Zhang et al., 2019
110	*TRPM6*	140803	Transient receptor potential cation channel subfamily M member 6	9q21.13	Saraç et al., 2016
111	*TXN2*	25828	Thioredoxin 2	22q12.3	Wen et al., 2009
112	*TYMS*	7298	Thymidylate synthetase	18p11.32	Martinez et al., 2009; Shaw et al., 2009
113	*UCP2*	7351	Uncoupling protein 2	11q13.4	Volcik et al., 2003
114	*VANGL1*	81839	VANGL planar cell polarity protein 1	1p13.1	Bartsch et al., 2012
115	*VANGL2*	57216	VANGL planar cell polarity protein 2	1q23.2	Kibar et al., 2011
116	*ZIC2*	7546	Zic family member 2	13q32.3	Klootwijk et al., 2004
117	*ZIC3*	7547	Zic family member 3	Xq26.3	Klootwijk et al., 2004

### SB Network Architecture Reveals Hierarchical Scale-Free Features

To construct the SB Protein-Protein Interaction (PPI) network, the candidate genes of SB listed in Table 1, were fed into STRING as seed genes. 116 of our seed genes got incorporated in the constructed primary network composed of 1,116 nodes and 40,886 edges, leaving *TRPM6*, which failed to make its way into the network. To gain structural insights into the primary PPI network of SB, we examined features of its topology or architecture i.e., probability of degree distribution P(k), clustering coefficient C(k), neighborhood connectivity C_*N*_(k), and centrality measurements. To find if the network has the scale-free fractal attribute and is hierarchical, the topological properties of the network must obey power-law behaviors as a function of degree *k* (Barabási and Albert, 1999) and it can be written as;

$$\text{Topological Property (TP)}∼{\text{k}}^{\wedge }\text{exp},$$

Where, degree distribution, neighborhood connectivity, clustering coefficient, betweenness centrality, closeness centrality, and eigenvector centrality are the topological properties and *γ*, β, α, μ, δ, *O* are their *exp* (exponents), respectively.

**FIGURE 1:**
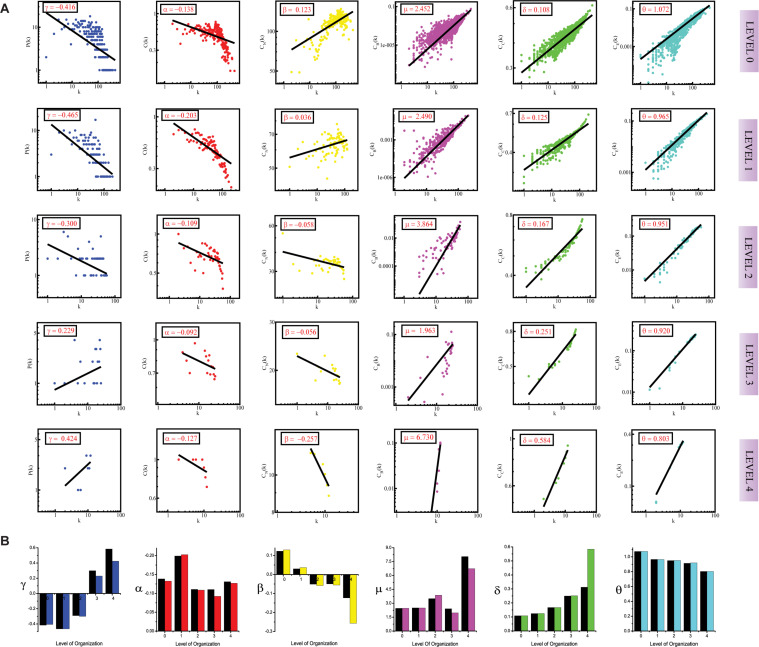
**(A)** showing probability of degree distribution *P*(*k*), clustering coefficient *C*(*k*), neighborhood connectivity *C*_*N*_(*k*), betweenness centrality *C*_*B*_(*k*), closeness centrality *C*_*C*_(*k*), and eigenvector centrality *C*_*E*_(*k*) as a function of degree (*k*) for primary original network (level 0) and *TNIP1* motif knockout networks at different levels of the organization (level 1–4). **(B)** showing changes in the values of the topological properties’ exponents of the *TNIP1* motif knockout networks (colors corresponding to the ones used in the topological properties plots i.e., blue for *P*(*k*), red for *C*(*k*), yellow for *C*_*N*_(*k*), magenta for *C*_*B*_(*k*), green for *C*_*C*_(*k*) and turquoise for *C*_*E*_(*k*)) compared with the topological properties’ exponents of the corresponding original networks (black) at different levels of the organization. γ, α, β, μ, δ, and O are the exponents of the degree distribution, clustering coefficient, neighborhood connectivity, betweenness centrality, closeness centrality, and eigenvector centrality, respectively.

**FIGURE 2:**
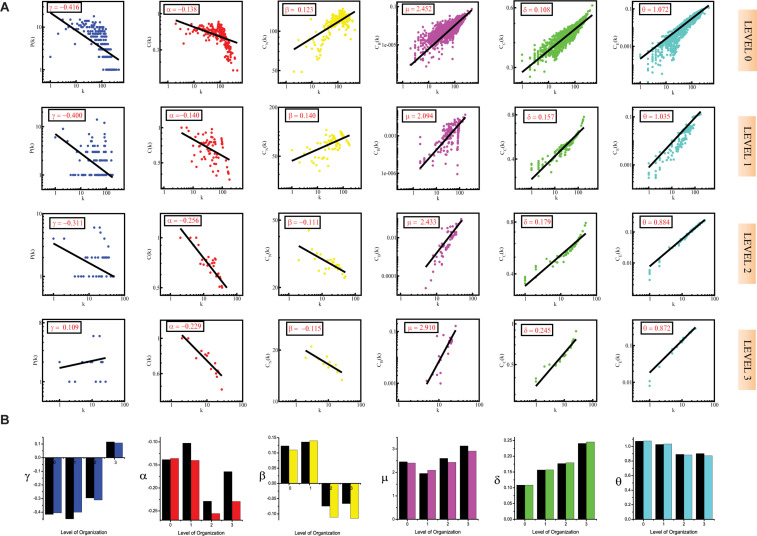
**(A)** showing probability of degree distribution *P*(*k*), clustering coefficient *C*(*k*), neighborhood connectivity *C*_*N*_(*k*), betweenness centrality *C*_*B*_(*k*), closeness centrality *C*_*C*_(*k*), and eigenvector centrality *C*_*E*_(*k*) as a function of degree (*k*) for primary original network (level 0) and *TNRC6B* motif knockout networks at different levels of the organization (level 1–3). **(B)** showing changes in the values of the topological properties’ exponents of the *TNRC6B* motif knockout networks [colors corresponding to the ones used in the topological properties plots i.e., blue for *P*(*k*), red for *C*(*k*), yellow for *C*_*N*_(*k*), magenta for *C*_*B*_(*k*), green for *C*_*C*_(*k*), and turquoise for *C*_*E*_(*k*)] compared with the topological properties’ exponents of the corresponding original networks (black) at different levels of the organization. γ, α, β, μ, δ, and O are the exponents of the degree distribution, clustering coefficient, neighborhood connectivity, betweenness centrality, closeness centrality, and eigenvector centrality, respectively.

**FIGURE 3:**
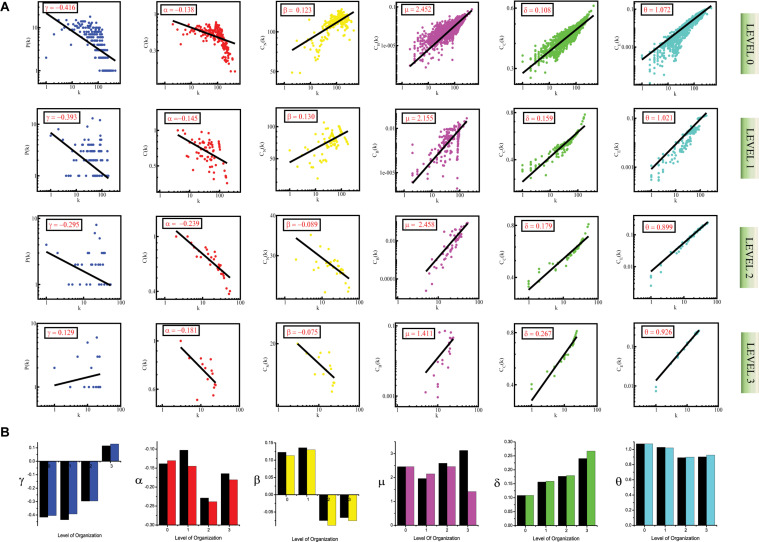
**(A)** showing probability of degree distribution *P*(*k*), clustering coefficient *C*(*k*), neighborhood connectivity *C*_*N*_(*k*), betweenness centrality *C*_*B*_(*k*), closeness centrality *C*_*C*_(*k*), and eigenvector centrality *C*_*E*_(*k*) as a function of degree (*k*) for primary original network (level 0) and *TRDMT1* motif knockout networks at different levels of the organization (level 1–3). **(B)** showing changes in the values of the topological properties’ exponents of the *TRDMT1* motif knockout networks [colors corresponding to the ones used in the topological properties plots i.e., blue for *P*(*k*), red for *C*(*k*), yellow for *C*_*N*_(*k*), magenta for *C*_*B*_(*k*), green for *C*_*C*_(*k*) and turquoise for *C*_*E*_(*k*)] compared with the topological properties’ exponents of the corresponding original networks (black) at different levels of the organization. γ, α, β, μ, δ, and O are the exponents of the degree distribution, clustering coefficient, neighborhood connectivity, betweenness centrality, closeness centrality, and eigenvector centrality, respectively.

We followed the standard statistical fitting technique put forward by Clauset et al. (2009) to verify that the features of the graph’s architecture follow power-law behavior. All statistical *P*-values for all data sets, calculated against 2,500 random samplings, are found to be larger than a critical value 0.1, and goodness of fits are found to be less than and equal to 0.33. The data points of all the topological parameters are found to fit power law when plotted against the degree *k* of the SB network (First row labeled as Level “0” in Figures 1A, 2A, 3A). The straight line in the graphs represents the non-linear curve-fitting on the formula *Y* = a0^∗^x^a1. Here a1 is the coefficient of the topological properties and we plot degree (*k*) on the *X*-axis. If these data fit well as stated by Al Barabasi et al., it is concluded that the network is scale-free and is hierarchical. In our study, it was seen that the topological properties of the network obey the power-law, thus, we conclude that the SB network is hierarchical and has scale-free fractal attributes. The values of the power-law exponents for each of the topological properties of the complete network were calculated:

(12) $$\left(\begin{array}{c} P\\ C\\ C_{N} \end{array}\right)∼\left(\begin{array}{c} K^{-\gamma }\\ K^{-\alpha }\\ K^{+\beta } \end{array}\right);\left(\begin{array}{c} \gamma_{0}\\ \alpha_{0}\\ \beta_{0} \end{array}\right)\to \left(\begin{array}{c} 0.416\\ 0.138\\ 0.123 \end{array}\right)$$

The values of the exponents of P(k), C(k), and C_*N*_(k), i.e., *γ*, α, and β, lead to the conclusion that the network though not having a strong hierarchy, still falls into the category of a weak hierarchical scale-free network, reflecting the presence of well-defined successive interconnected communities with meagerly distributed hubs (nodes with a high degree of interaction) in the network (Pastor-Satorras et al., 2001; Ravasz et al., 2002; Nafis et al., 2015). The negative values of *γ* (*γ* < 2) and α, indicate the hierarchical nature of the SB network. The negative value of α means that as *k* is increasing, C(k) is decreasing, indicating that nodes with a high degree have a low tendency to cluster, further showing a hierarchy of hubs, in which the most densely connected hub is linked to a small fraction of all other nodes. The power-law obeyed by P(k) is also a sign of the scale-free nature of the SB network since scale-free networks are defined by a power-law degree distribution, the negative value of *γ* means that a minor section of the nodes exhibits a high degree with most of the nodes having a low degree which is in accordance with the definition of a scale-free network (Barabási and Albert, 1999). The positive value of β indicates the assortative nature of the SB network, which means that edges between heavily connected nodes predominate in the network to regulate the latter (Pastor-Satorras et al., 2001).

Centrality measurements, namely betweenness centrality C_*B*_(k), closeness centrality C_*C*_(k), and eigenvector centrality C_*E*_(k) are also observed to exhibit power-law or fractal behavior:

(13) $$\left(\begin{array}{c} C_{B}\\ C_{C}\\ C_{E} \end{array}\right)∼\left(\begin{array}{c} K^{\mu }\\ K^{\delta }\\ K^{\theta } \end{array}\right);\left(\begin{array}{c} \mu_{0}\\ \delta_{0}\\ \theta_{0} \end{array}\right)\to \left(\begin{array}{c} 2.452\\ 0.108\\ 1.072 \end{array}\right)$$

The positive values of the exponents *μ*, *δ*, and θ of the three distributions C_*B*_(k), C_*C*_(k), and C_*E*_(k), respectively, also show that the network exhibits hierarchical scale-free or fractal features (Bonacich, 1987). The positive values of these exponents mean that C_*B*,_ C_*C*_, and C_*E*_ when plotted against degree *k*, increase as *k* increases. The increasing value of C_*B*_ as *k* increased indicates that nodes with a high degree have high C_*B*,_ thus, these larger hubs have major influence over the information transmission in the network than the nodes with a low degree. Similarly, direct proportionality between C_*C*_ and *k* points toward the high C_*C*_ of the hubs further indicating that these high-degree nodes are the quick spreader of the information in the network. C_*E*_ is also in favor of highly connected nodes as reflected by the positive value of θ, showing that nodes with a high degree have high C_*E*_ as well, thus indicating that these nodes are more influential in the network on account of their power of spreading information in the network. The positive value of θ signifies the connectedness between the high degree nodes, this is in agreement with the assortative mixing in the network.

Thus, through meticulous study of these topological properties, the SB network was found to self-organize into a scale-free fractal state, having a weakly hierarchical organization.

### Key Regulators Uncovered Through Clustering and Tracing

**FIGURE 4:**
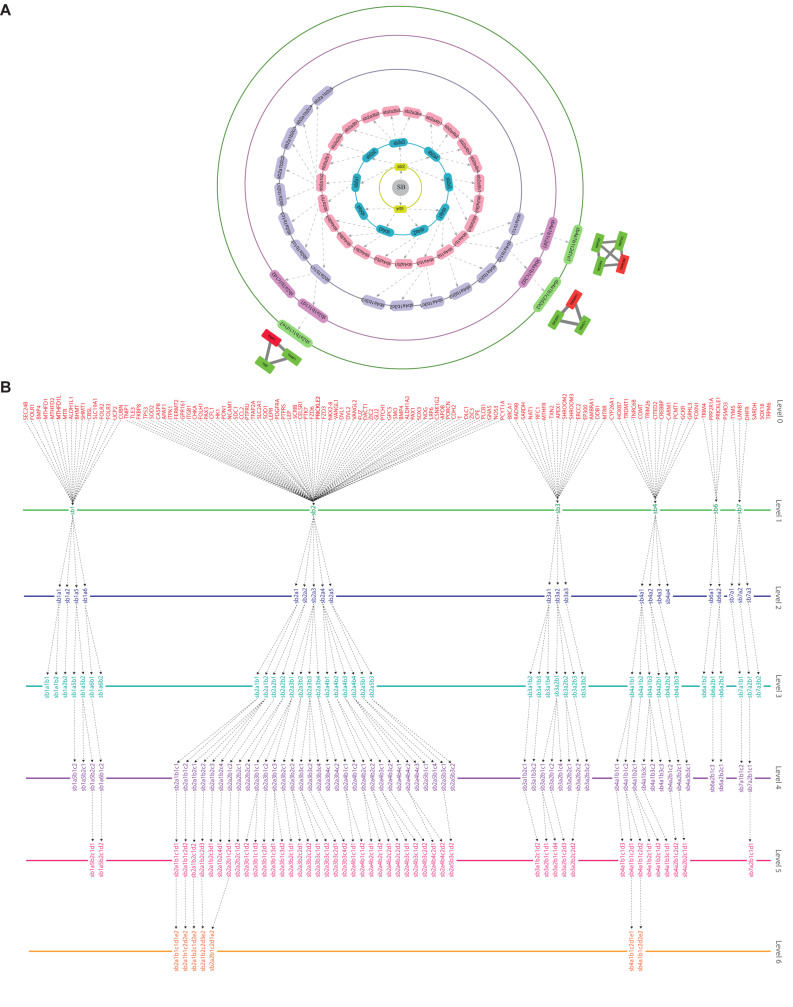
**(A)** illustration of the hierarchical organization of the SB network into 6 different levels that resulted after clustering. The gray-colored circle in the center represents the primary network of SB which is level 0, the primary network got divided into seven modules after clustering making it the next level of hierarchy i.e., level 1. Each subsequent circle represents the next level of the SB network’s organization and arrows indicate submodules emerging from the previous module. **(B)** tracing of the seed genes through different hierarchical levels of the SB network starting from the main network (SB) i.e., level 0 up to the motif level i.e., level 6.

**FIGURE 5:**
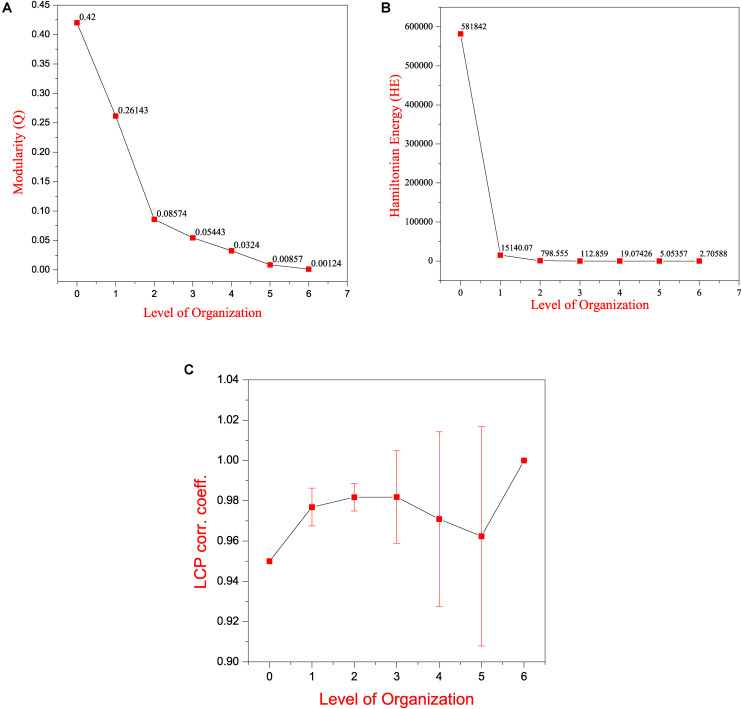
Features of the SB network **(A)** modularity (Q) plotted against levels of the hierarchical organization of the SB network. **(B)** Hamiltonian Energy (HE) plotted against levels of the hierarchical organization of the SB network. **(C)** variation in the calculated average LCP-corr coefficient as a function of levels of the hierarchical organization of the SB network.

**FIGURE 6:**
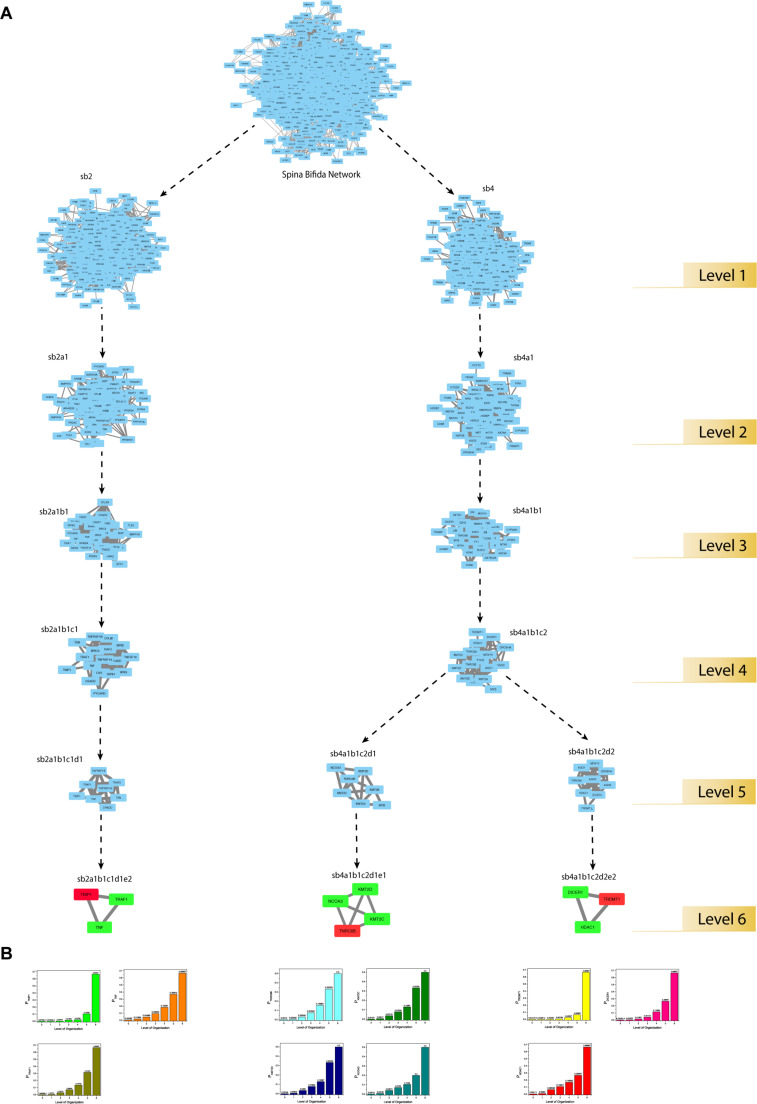
**(A)** modular path of the KRs starting from the main network to the motif level. The seed-gene-KRs are shown in red and their interacting-partner-KRs are shown in green. **(B)** KRs probability distribution as a function of levels of the hierarchical organization of the SB network.

Through clustering using Newman and Girvan’s algorithm, the network got divided into communities and sub-communities distributed in six hierarchical levels (Figure 4A). It appeared that both modularity (Q) and Hamiltonian Energy (HE) decrease from top to bottom organization when plotted against the level of organization (Figures 5A,B). When the seed genes were traced from top to bottom organization of the SB network through these levels of hierarchy, only three of them were found to reach the last sixth level (motif level) of the SB network, namely, *TNIP1*, *TNRC6B*, and *TRDMT1*. These three seed genes along with their interacting partners in the last level of the network organization, namely, *TNF* and *TRAF1* (interacting partners of *TNIP1*); *KMT2C*, *KMT2D*, and *NCOA3* (interacting partners of *TNRC6B*); and *DICER1* and *HDAC1* (interacting partners of *TRDMT1*) were revealed to be the KRs of the SB network, the criterion being their ability to make triangular motifs [defined by G(3, 3)] (Figure 6A) and their presence at every topological level (Figure 4B). This is in agreement with the definition of KRs, according to which, KRs are the genes/proteins which are deeply rooted from the top to bottom organization of the network. These KRs are considered to be the backbone in maintaining a network’s stability as they capacitate the network to combat any unacceptable alterations in it. Five more of our seed genes namely, *ITPK1*, *FKBP8*, *TLE3*, *FOLH1*, and *TP53*, reached the sixth level but they could not be considered as KRs because of their shortcoming to form triangular motifs.

The regulating ability of each of the KRs was estimated by defining the probability *P*_*KR*_(*x*^*l*^) of the KRs to regulate the networks at different levels (Figure 6B). *P*_*KR*_(*x*^*l*^) gives the proportion of the edges count (*x*), the KR has in the network/community/sub-community compared to the total edges count (*E*) present in the network/community/sub-community at *l* level of organization:

(14) $$P_{KR}\left(x^{\left[l\right]}\right)=\frac{x^{\left[l\right]}}{E^{\left[l\right]}}$$

*P*_*KR*_(*x*^*l*^) of all the KRs was found to be increasing with an increase in the level of organization. This suggests that the regulatory role of the KRs is even more powerful at deeper levels, thus these behave as active workers at the grassroots level.

### Low Popularity Maintained by the Identified Key Regulators

**FIGURE 7:**
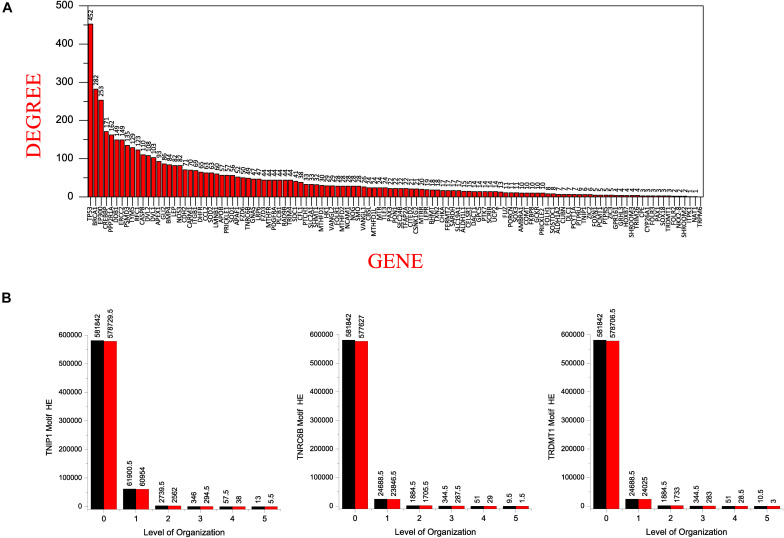
**(A)** showing the seed genes in the decreasing order of their degree with *TP53* having the highest degree. **(B)** comparison of the Hamiltonian Energy (HE) of the original (black) and the corresponding motifs knockout networks (red) at different levels of the hierarchical organization of the SB network.

All of our KRs had a somewhat low degree in the main network which dramatically changed in the subsequent levels of the organization. Of our six KRs, *TNF* has the popularity-rank (rank on the basis of degree) of 42 in the main network, decreasing as we move to *HDAC1*(70), *NCOA3*(374), *KMT2D*(559), *KMT2C*(612), *TNRC6B*(652), *DICER1*(735), *TRAF1*(806), *TNIP1*(1072), and *TRDMT1*(1099). Surprisingly, the first 41 leading hubs at the complete network-level, failed to make their way to the last level of the organization leading us to infer that the popularity-rank of leading hubs does not assure the emergence of the hubs as KRs (Figure 7A).

### Emergence of Low Degree Nodes Accompanied by High Degree Node as Key Regulators

In a network, when a node’s degree is low, the node gains what strength it has from its neighbors and thus the influence it has over the network is a function of its neighbors’ degree. In addition, a low-degree bridge node, connecting two high-degree nodes, is very important in a network despite its lower degree (Liu et al., 2016). Thus, a node’s degree is not the sole determinant of its essentiality, rather, it depends on the topological position of that node. This is reflected in our results, where, the three seed genes, i.e., *TNIP1*, *TNRC6B*, and *TRDMT1* have a relatively low degree compared to their interacting partners in the primary network, but all three of these genes, as well as their interacting partners, are found out to be the KRs of the SB network based on their ability to make it to the last level of the network organization. *TNIP1* which has quite a low degree of 6, formed a motif in the last level of the organization with *TNF* and *TRAF1* which have degree 197 and 31, respectively; likewise, *TNRC6B* which has a degree of 49 formed motifs with *KMT2C*, *KMT2D*, and *NCOA3* which have degree 52, 59, and 87, respectively; and *TRDMT1* which has such a low degree of 3 formed a motif with *DICER1* and *HDAC1* which have degree 40 and 168, respectively, in the primary network. This shows that the three low degree seed genes were regulated by their relatively high degree interacting partners from top to bottom organization of the SB network indicating that the until now unverified interacting partners could be directly or indirectly related to the pathophysiology of SB in humans.

### Perturbations Driven by the Knockout of the Motifs Consisting of the Key Regulators

The hierarchical topology of the SB network saves it from breaking down completely after the removal of the KRs from it, owing to the strong self-organizing nature of a hierarchical network (Ravasz et al., 2002). However, when KRs are removed from a network it may lead to certain local and global disturbances in the network which will propagate through various levels of the hierarchy starting from top to bottom or bottom to top, causing changes in the network’s topology.

Based on the property of a module that its function is different from other modules and that its nodes have more relations to each other than to members of other modules (Dong and Horvath, 2007), we knocked out the KRs-containing modules/motifs that were found in the last level of the SB network organization from the main network-level till the last level and then studied the changes in the topological properties of the SB network due to the motifs knockout to comprehend the regulatory role of the KRs in the same combination as they were found in the motifs.

In the case of the *TNIP1* motif (consisting of *TNIP1, TNF*, and *TRAF1 KRs)* the network/modules/submodules keep adapting themselves functionally to cope up with the removal of this motif till the fourth level, from the fifth level its knock-out causes the sub-communities it is present in, to almost fall apart. Whereas, speaking of *TNRC6B* motif (consisting of *TNRC6B, KMT2C*, *KMT2D*, and *NCOA3*) and *TRDMT1* motif (consisting of *TRDMT1, DICER1*, and *HDAC1*) the submodules from the fourth level itself lose their ability to adapt functionally in response to the loss of these motifs and thus break down. In all the cases a considerable change in the network’s topological parameters was noticed, however, somehow the network reorganizes itself thereby proving to have an error tolerance. In all the motifs knockout networks, the value of *γ* is found to turn positive in the deeper organization levels, reflecting the loss of scale-free topology of the communities in the deeper levels of the organization (Barabási and Albert, 1999). For all the motifs, a consistent pattern is seen in the value of α in the knockout networks, an initial increase in the value of α indicates increased compactness of the networks so as to rescue the networks from falling apart but with a decrease in the level of organization, α is also found to be decreasing which points toward decreasing compactness of the communities (Ravasz and Barabási, 2003). The value of β becoming negative in the deeper levels of the organization shows that the networks have become disassortative in nature (Pastor-Satorras et al., 2001). The value of *μ* is noted to be decreasing in the deeper levels of the organization upon knock-out of the motifs, indicating the decreasing significance of the remaining hubs’ regulatory roles in the networks (Borgatti and Everett, 2006). The increase in the value of *δ* reveals faster information processing within the network upon removal of the motifs so as to reorganize the perturbed network and rescue it from falling apart (Canright and Engø-Monsen, 2004). Lastly, the value of θ which is found to be decreasing, shows that information transmission is reduced because the KRs-containing motifs are removed (Canright and Engø-Monsen, 2006; Figures 1A, 2A, 3A).

In all the motifs knockout experiments, the exponent values of all of the topological properties showed drastic changes in lower levels of the hierarchy, signifying that local perturbation increases as we go to deeper levels starting from top to bottom in the network (Figures 1B, 2B, 3B).

By calculating and comparing the Hamiltonian energy (HE) of the original network/community/sub-community with the corresponding modified network/community/sub-community that resulted after the motifs knockout experiments, we observed a slight reduction in HE in the knockout networks at each level of organization (Figure 7B). This demonstrates that the KRs knockout experiments result in loss of wiring/rewiring energy at all levels of the network organization.

### Functional Enrichment and Pathway Enrichment of the SB Network’s Seed Genes

We identified different molecular functions, biological pathways, and cellular components as well as KEGG pathways in which the seed genes (candidate genes for SB) are significantly enriched using DAVID functional annotation tool, listed in Table 2. It was found that most of the seed genes are enriched in neural tube closure, folic acid-binding, and regulation of transcription. These functions and pathways may serve important roles in the pathogenesis of SB.

**TABLE 2:** Functional and pathway enrichment of the SB network’s seed genes.

Category	S. no.	GO Term	Seed Gene Count	Benjamini-adjusted *P*-Value
Biological Process	1.	Neural tube closure	21	1.46E-23
	2.	Positive regulation of transcription from RNA polymerase II promoter	19	0.006187585
	3.	Positive regulation of transcription, DNA-templated	18	0.0000199
	4.	Negative regulation of transcription from RNA polymerase II promoter	16	0.006187585
	5.	Oxidation-reduction process	15	0.003218669
Cellular Component	1.	Cytoplasm	57	0.000444
	2.	Cytosol	46	0.000033
	3.	Caveola	6	0.003341129
Molecular Function	1.	Beta-catenin binding	7	0.003585258
	2.	Folic acid binding	6	0.0000059
	3.	Damaged DNA binding	6	0.006137571
	4.	Wnt-protein binding	5	0.006137571
KEGG Pathway	1.	Pathways in cancer	17	1.33E-04
	2.	Wnt signaling pathway	14	1.58E-07
	3.	One carbon pool by folate	9	2.46170877161806E-09
	4.	Basal cell carcinoma	9	5.06271234450916E-06
	5.	Melanogenesis	8	0.003208

### Evidence of Self-Organization: Local-Community-Paradigm (LCP) Approach

Local-community-paradigm correlation of all the communities/sub-communities at each level of the hierarchy was computed. The average values of the LCP-corr for all the modules at each level are found to be greater than 0.95 and these values do not vary with an error bar (modules with zero LCP-corr were excluded while calculating the average) (Figure 5C). The high values of the calculated LCP-corr for all the modules/sub-modules reflect strong compactness of these modules/submodules, this means that these modules/sub-modules are composed of tightly connected nodes, which strongly favors the preservation of the network properties and adaptation of the network against any unfavorable changes in it to prevent the network from breaking down.

### Rich-Club Organization in SB Network

**FIGURE 8:**
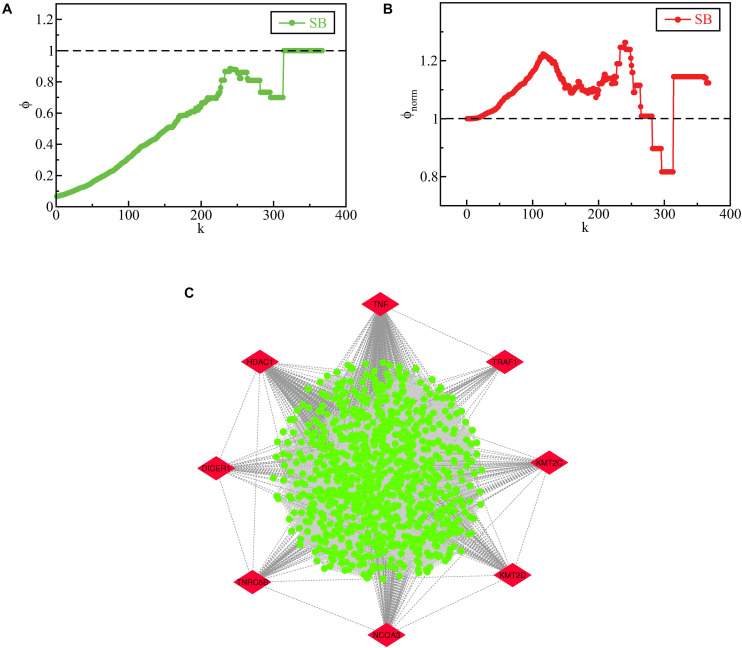
**(A)** raw rich-club coefficient (ϕ) of the SB network as a function of degree *k*. **(B)** normalized rich-club coefficient (ϕ_*norm*_) of the SB network as a function of degree *k*. **(C)** rich club nodes of the SB network, the KR genes which emerged as rich-club nodes are highlighted in red.

When a PPI network related to a disease exhibits a rich-club formation, it evinces the existence of a pathological powerhouse within the network, composed of the most influential components which have to their credit of providing the network robustness and stability (Alawieh et al., 2015). We evaluated the existence of a rich-club structural ordering in the SB network using brainGraph package in R and the network was found to exhibit a rich club structural ordering as indicated by an increasing rich-club coefficient ϕ(*k*) as *k* increased (Figure 8A). Further, the relevance of the uncovered rich-club organization was examined by assessing the normalized ϕ(*k*) (ϕ_*norm*_(*k*)) (Figure 8B). ϕ_*norm*_(*k*) was calculated for the lowermost degree (1) to the third-highest degree (364) in the SB network with a rich-club showing between degrees 25 to 364. The degree found to have the highest ϕ_*norm*_(*k*) is 240 and the node having the highest ϕ_*norm*_(*k*) corresponding to the degree 240 is *CTNNB1*. The subnetwork of nodes with degrees corresponding to the normalized rich-club coefficient ≥1.1 is shown in Figure 8C and defined as the rich-club nodes. Besides being the KRs of the SB network, *TNF*, *TRAF1*, *KMT2C*, *KMT2D*, *NCOA3*, *TNRC6B*, *DICER1*, and *HDAC1* emerged as rich-club nodes as well.

## Discussion

A dearth of knowledge about the genetic etiology of Spina bifida (SB) demands exploration of its genetic aspects. Though several candidate genes for SB have been reported in recent years, however, the potential mechanism underlying SB development remains unclear. To the best of our knowledge, our *in silico* study is the first attempt toward investigating the Protein-Protein Interaction (PPI) network of SB and identifying the Key Regulators (KRs) of SB through topological and rich-club analysis that regulate the whole network.

The study of the topological properties of our primary network (SB) consisting of 1,116 nodes and 40,886 edges, suggests that the network follows a weak hierarchical and scale-free fractal nature. The hierarchical topology indicates the two-tier organization of the network with one level representing local clustering of mostly low degree nodes into well-defined successive communities or modules and the other level representing more global connectivity in which the hubs serve as higher-order communication points between the interconnected communities. The fractal state of the network signifies a self-similar organization of the network and scale-free nature has to its credit of making the network stable. These topological properties allow efficient information processing within the network. The topological properties analysis of the SB network was essential to know the behavior of the network and to validate that the network is following the criteria of a scale-free and hierarchical network which further was requisite for the identification of the most significant KRs of the network. After clustering, the self-organizing behavior of the SB networks was investigated through the LCP technique which led us to infer that the networks sustain self-organization based on their dynamic nature and are tightly packed with efficient information processing. Through Gene Ontology and pathway enrichment analysis it was found that the seed genes are enriched in neural tube closure and regulation of transcription. These functions and pathways may serve important roles in the pathogenesis of SB.

Key regulators are the regulatory entities within a network that are sufficient to induce a complete complex developmental pathway. Out of our 117 seed genes, 3 genes, namely, *TNIP1*, *TNRC6B*, and *TRDMT1*, were found to reach the motif level of the SB network hierarchical organization. Though all the seed genes are involved in SB, however, the above-mentioned three seed genes are specifically considered to be the backbone of the SB network with regard to maintaining the network’s stability based on their ability to make it to the grassroots level. Also, the importance of their interacting partner genes, namely, *TNF* and *TRAF1* (interacting partners of *TNIP1*); *KMT2C*, *KMT2D*, and *NCOA3* (interacting partners of *TNRC6B*); and *DICER1* and *HDAC1* (interacting partners of *TRDMT1*) cannot be ruled out as they formed motifs with these seed genes in the last level of the hierarchical organization of the SB network which means that these seed genes work in combination with their interacting partners and hence these genes, as well as their interacting partners, were revealed to be the KRs of the network. Since there is no direct strong evidence in the pre-existing literature for these interactor genes’ involvement in SB to date, our study is indicating the potential role of these genes in SB.

The *TNF* (tumor necrosis factor) gene encodes a protein named TNF-α which is an inflammatory cytokine. This cytokine is responsible for a diverse range of signaling events within cells through the NF-κB pathway that ultimately leads to necrosis or apoptosis (Idriss and Naismith, 2000). TNF-α induces TNFAIP3, tumor necrosis factor-α-induced-protein 3 which is also known as A20, TNFAIP3 interacts with TNIP1 gene-encoded TNFAIP3 interacting protein 1 (TNIP1 protein), TNFAIP3 and TNIP1 then in conjunction regulate NF-κB dependent gene expression negatively (Heyninck et al., 1999). When NF-κB is active, it turns on the expression of those genes that keep the cell proliferating. So, a constitutively active state of NF-κB leads to various types of human tumors. Since TNIP1 is a negative regulator of the NF-κB pathway, so when the TNIP1-TNFAIP3 complex inhibits NF-κB, anti-apoptotic genes are not activated by NF-κB and hence apoptosis happens. TRAF1 gene-encoded tumor necrosis factor receptor-associated factor 1 (TRAF1) is an adapter in signal transduction and has roles in apoptotic processes in different cell types as well as in immunity. TRAF1 is one of the anti-apoptotic genes that are induced by NF-κB and its encoded protein, TRAF1, is responsible for resistance to apoptosis downstream of CD30 which is a protein of the tumor necrosis factor family (Zhang et al., 2014; Edilova et al., 2018). Hence, defects in all these three genes *TNF, TRAF1*, and *TNIP1* are related to the formation of cancer in one way or the other. Also, a clear positive association has been shown in a study between SB and pediatric cancer pointing toward an overlap between genes impacting both development and malignancy. Therefore, these genes may have important roles in SB (Johnson et al., 2017).

NCOA3, Nuclear receptor coactivator 3, a member of the p160 family of coactivators facilitates the upregulation of gene expression by functioning as a transcriptional coactivator protein. It is recruited to the DNA promotion sites by ligand-activated nuclear receptors, where it acylates histones making the downstream DNA more accessible to transcription (Zhang et al., 2018). Like NCOA3, KMT2C (lysine methyltransferase 2C) and KMT2D (lysine methyltransferase 2D) proteins also modify histones by functioning as H3K4me1/2 methyltransferases, these two proteins have pivotal roles in embryonic morphogenesis, central nervous system development, and post-natal survival (Lavery et al., 2020). TNRC6B, Trinucleotide Repeat Containing Adaptor 6B protein participates in RNA-mediated gene silencing through both miRNA-dependent translational repression and siRNA-dependent endonucleolytic cleavage of complementary mRNAs by argonaute family proteins. It needs to be mentioned here that the TNRC6B paralog TNRC6A protein has been found to interact with histone-modifying complexes (Hicks et al., 2017). Hence, it is evident that these four genes, namely, *NCOA3*, *KMT2C*, *KMT2D*, and *TNRC6B* are interrelated to each other in their function. Also, it has been reported that the *TNRC6B* gene shows an association with myelomeningocele in the Mexican American population (Hebert et al., 2020). Therefore, these genes might have roles in SB.

HDAC1 (Histone Deacetylase 1) protein localizes into the nucleus and regulates eukaryotic gene expression via deacetylation of all the four core histones. It forms a complex with retinoblastoma tumor-suppressor protein and controls cell proliferation and differentiation. Also, in conjunction with metastasis-associated protein-2, it deacetylates and destabilizes p53 and modulates its effect on cell growth and apoptosis (Milazzo et al., 2020). The *DICER1* gene encodes the endoribonuclease Dicer protein of the ribonuclease III family. MicroRNAs (miRNAs) are created by the Dicer endoribonuclease protein, which are known to control gene expression by binding to specific mRNAs in order to inhibit the access of ribosomes to these mRNAs and their subsequent translation (Robertson et al., 2018). *TRDMT1* gene codes for a methyltransferase, tRNA aspartic acid methyltransferase 1, that methylates as the name suggests, a specific RNA molecule, the aspartic acid transfer RNA (tRNAAsp). It has been reported in a study carried out in a Dutch population that the A allele of the disease-associated rs2295809 polymorphism in *TRDMT1* was associated with an increased RBC folate in the control population, which is in accordance with its risk-reducing effect observed in this study (Goll et al., 2006; Franke et al., 2009). So it is quite evident that one thing is common in these three genes that all of these are gene expression regulators and one of these i.e., *TRDMT1* has been shown to be involved in SB. Therefore, these genes can be considered to have roles in the occurrence of SB.

Low popularity exhibited by all of the KRs in the main network leads us to infer that these KRs outrun the leading hubs in their significance to propagate signals through the different hierarchical levels of the network in order to conserve the network’s stability and intrinsic properties. Motifs knockout experiments imparted maximum local perturbation in the deeper levels of the hierarchy but their removal did not trigger off the networks to break down, this substantiates the self-organizing ability of the networks that somehow kept harmonizing and coping with the motifs (consisting of the KRs) knockout.

The essentiality of the identification of rich-club nodes in a network lies in the fact that these nodes create a sub-structure within the network that increases the stability of the whole network and improves the efficacy of information transmission among high degree nodes. Through rich-club analysis, it was found that *TNF*, *TRAF1*, *KMT2C*, *KMT2D*, *NCOA3*, *TNRC6B*, *DICER1*, and *HDAC1*, apart from being the KRs of the SB network, also emerged as rich-club nodes. This finding further adds significance to our study as these KRs have proven out to be involved in the formation of the rich-club organization within the SB network to boot. This evinces that these key genes take part in becoming a transit backbone of the network and increase the network’s stability as a whole thus providing robustness to the network.

## Conclusion

In this study, we have employed graph theory to identify important functional modules/motifs consisting of the novel key regulators of SB. While our study puts forward the most important genes from among the candidate seed genes of SB, namely, *TNIP1*, *TNRC6B*, and *TRDMT1*, it also uncovers novel genes, namely, *TNF*, *TRAF1*, *KMT2C*, *KMT2D*, *NCOA3*, *DICER1*, and *HDAC1* which can be directly or indirectly related to SB as these genes have been found to regulate the above-mentioned seed genes till the last level of the SB network, hence all of these genes are concluded to be the KRs of SB. As it is well known that the proteins in the same module share the same function so if all the KRs of a single module/motif are targetted together, they can serve as biomarkers for the diagnosis of SB. Since SB has multiple etiology and mechanisms so the combination of several biomarkers may have high diagnostic accuracy for SB compared to using a single biomarker. Experimental validation of these hypotheses would confirm the credibility of the identified KRs which may serve as the key targets for therapeutic interventions and as putative prognostic biomarkers.

## Data Availability Statement

The original contributions presented in the study are included in the article/supplementary material, further inquiries can be directed to the corresponding authors.

## Author Contributions

RI and NT conceived the study design instructed on data analysis. NT curated data and performed statistical and network analysis and prepared the figures of the numerical results. NT and AF analyzed and interpreted the simulated results. NT wrote the manuscript. SYA, SAA, AAJ, AF, ST, NA, and RI reviewed the manuscript. RI and NA jointly supervised the study and approved the final draft. All authors contributed to the article and approved the submitted version.

## Conflict of Interest

The authors declare that the research was conducted in the absence of any commercial or financial relationships that could be construed as a potential conflict of interest.

## Footnotes

1 http://string-db.org/

2 http://igraph.sf.net

## References

[B1] Agopian A. J. Bhalla A. D. Boerwinkle E. Finnell R. H. Grove M. L. Hixson J. E. (2013). Exon sequencing of PAX3 and T (brachyury) in cases with spina bifida. *Br. Defects Res. A. Clin. Mol. Teratol.* 97 597–601. 10.1002/bdra.23163 23913553 PMC3877942

[B2] Alawieh Z. Sabra M. Sabra L. Tomlinson S. Zaraket F. A. (2015). A rich-club organization in brain ischemia protein interaction network. *Sci. Rep.* 5:13513. 10.1038/srep13513 26310627 PMC4550934

[B3] Albert R. Barabási A.-L. (2002). Statistical mechanics of complex networks. *Rev. Mod. Phys.* 74 47–97. 10.1103/RevModPhys.74.47

[B4] Ali S. Malik M. D. Z. Singh S. S. Chirom K. Ishrat R. Singh R. K. B. (2018). Exploring el key regulators in breast cancer network. *PLoS One* 13:e0198525. 10.1371/journal.pone.0198525 29927945 PMC6013121

[B5] Amorosi S. D’Armiento M. Calcagno G. Russo I. Adriani M. Christiano A. M. (2008). FOXN1 homozygous mutation associated with anencephaly and severe neural tube defect in human athymic Nude/SCID fetus. *Clin. Genet.* 73 380–384. 10.1111/j.1399-0004.2008.00977.x 18339010

[B6] Barabási A.-L. Albert R. (1999). Emergence of scaling in random networks. *Science* 286 509–512. 10.1126/science.286.5439.509 10521342

[B7] Barabási A. L. Gulbahce N. Loscalzo J. (2011). Network medicine: a network-based approach to human disease. *Nat. Rev. Genet.* 12 56–68. 10.1038/nrg2918 21164525 PMC3140052

[B8] Barrat A. Barthélemy M. Pastor-Satorras R. Vespignani A. (2004). The architecture of complex weighted networks. *Proc. Natl. Acad. Sci. U.S.A.* 101 3747–3752. 10.1073/pnas.0400087101 15007165 PMC374315

[B9] Bartsch O. Kirmes I. Thiede A. Lechno S. Gocan H. Florian I. S. (2012). VANGL1 gene mutations in 144 slovakian, romanian and german patients with neural tube defects. *Mol. Syndromol.* 3 76–81. 10.1159/000339668 23326252 PMC3542939

[B10] Bassuk A. G. Muthuswamy L. B. Boland R. Smith T. L. Hulstrand A. M. Northrup H. (2013). Copy number variation analysis implicates the cell polarity gene glypican 5 as a human spina bifida candidate gene. *Hum. Mol. Genet.* 22 1097–1111. 10.1093/hmg/dds515 23223018 PMC3578410

[B11] Bauters M. Frints S. G. Van Esch H. Spruijt L. Baldewijns M. M. de Die-Smulders C. E. M. (2014). Evidence for increased SOX3 dosage as a risk factor for X-linked hypopituitarism and neural tube defects. *Am. J. Med. Genet. A* 164A 1947–1952. 10.1002/ajmg.a.36580 24737742

[B12] Beaudin A. E. Stover P. J. (2007). Folate-mediated one-carbon metabolism and neural tube defects: balancing genome synthesis and gene expression. *Birth Defects Res. Part C Embryo Today Rev.* 81 183–203. 10.1002/bdrc.20100 17963270

[B13] Bonacich P. (1987). Power and centrality: a family of measures. *Am. J. Sociol.* 92 1170–1182. 10.1086/228631

[B14] Borgatti S. P. Everett M. G. (2006). A Graph-theoretic perspective on centrality. *Soc. Netw.* 28 466–484. 10.1016/j.socnet.2005.11.005

[B15] Bosoi C. M. Ca V. Allache R. Trinh V. Q.-H. Marco P. D. Merello E. (2011). Identification and characterization of el rare mutations in the planar cell polarity gene PRICKLE1 in human neural tube defects. *Hum. Mutat.* 32 1371–1375. 10.1002/humu.21589 21901791 PMC3217084

[B16] Brandes U. (2001). A faster algorithm for betweenness centrality. *J. Math. Sociol.* 25 163–177. 10.1080/0022250X.2001.9990249

[B17] Brei T. Houtrow A. (2017). Spina bifida. *J. Pediatr. Rehabil. Med.* 10 165–166. 10.3233/PRM-170469 29154296

[B18] Brown K. S. Cook M. Hoess K. Whitehead A. S. Mitchell L. E. (2004). Evidence that the risk of spina bifida is influenced by genetic variation at the NOS3 locus. *Birt. Defects Res. A. Clin. Mol. Teratol.* 70 101–106. 10.1002/bdra.20002 15039923

[B19] Browne F. Wang H. Zheng H. (2018). Investigating the impact human protein-protein interaction networks have on disease-gene analysis. *Int. J. Mach. Learn. Cybern.* 9 455–464. 10.1007/s13042-016-0503-5

[B20] Cacciola A. Naro A. Milardi D. Bramanti A. Malatacca L. Spitaleri M. (2019). Functional brain network topology discriminates between patients with minimally conscious state and unresponsive wakefulness syndrome. *J. Clin. Med.* 8:306. 10.3390/jcm8030306 30841486 PMC6463121

[B21] Cadenas-Benitez N. M. Yanes-Sosa F. Gonzalez-Meneses A. Cerrillos L. Acosta D. Praena-Fernandez J. M. (2014). Association of neural tube defects in children of mothers with MTHFR 677TT genotype and abnormal carbohydrate metabolism risk: a case-control study. *Genet. Mol. Res. GMR* 13 2200–2207. 10.4238/2014.March.26.8 24737468

[B22] Cannistraci C. V. Alanis-Lobato G. Ravasi T. (2013). From link-prediction in brain connectomes and protein interactomes to the local-community-paradigm in complex networks. *Sci. Rep.* 3:1613. 10.1038/srep01613 23563395 PMC3619147

[B23] Canright G. Engø-Monsen K. (2004). Roles in networks. *Sci. Comput. Program.* 53 195–214. 10.1016/j.scico.2003.12.008

[B24] Canright G. S. Engø-Monsen K. (2006). Spreading on networks: a topographic view. *Complexus* 3 131–146. 10.1159/000094195

[B25] Cao X. Tian T. Steele J. W. Cabrera R. M. Aguiar-Pulido V. Wadhwa S. (2020). Loss of RAD9B impairs early neural development and contributes to the risk for human spina bifida. *Hum. Mutat.* 41 786–799. 10.1002/humu.23969 31898828 PMC7185173

[B26] Carter T. C. Pangilinan F. Troendle J. F. Molloy A. M. VanderMeer J. Mitchell A. (2011). Evaluation of 64 candidate single nucleotide polymorphisms as risk factors for neural tube defects in a large irish study population. *Am. J. Med. Genet. A* 155A 14–21. 10.1002/ajmg.a.33755 21204206 PMC3503244

[B27] Chen S. Zhang Q. Bai B. Ouyang S. Bao Y. Li H. (2017). MARK2/Par1b insufficiency attenuates DVL gene transcription via histone deacetylation in lumbosacral spina bifida. *Mol. Neurobiol.* 54 6304–6316. 10.1007/s12035-016-0164-0 27714636

[B28] Chen S.-J. Liao D.-L. Chen C.-H. Wang T.-Y. Chen K.-C. (2019). Construction and analysis of protein-protein interaction network of heroin use disorder. *Sci. Rep.* 9:4980. 10.1038/s41598-019-41552-z 30899073 PMC6428805

[B29] Chen Z. Kuang L. Finnell R. H. Wang H. (2018). Genetic and functional analysis of SHROOM1-4 in a Chinese neural tube defect cohort. *Hum. Genet.* 137 195–202. 10.1007/s00439-017-1864-x 29423651 PMC5876139

[B30] Clauset C. Shalizi R. Newman M. E. J. (2009). Power-law distributions in empirical data. *SIAM Rev.* 51 661–703.

[B31] Colizza V. Flammini A. Serrano M. A. Vespignani A. (2006). Detecting rich-club ordering in complex networks. *Nat. Phys.* 2 110–115. 10.1038/nphys209

[B32] Copp A. J. Adzick N. S. Chitty L. S. Fletcher J. M. Holmbeck G. N. Shaw G. M. (2015). Spina Bifida. *Nat. Rev. Dis. Primer* 1:15007. 10.1038/nrdp.2015.7 27189655 PMC4898641

[B33] Davidson C. M. Northrup H. King T. M. Fletcher J. M. Townsend I. Tyerman G. H. (2008). Genes in glucose metabolism and association with spina bifida. *Reprod. Sci.* 15 51–58. 10.1177/1933719107309590 18212354 PMC2592548

[B34] De Marco P. Calevo M. G. Moroni A. Merello E. Raso A. Finnell R. H. (2003). Reduced folate carrier polymorphism (80A→G) and neural tube defects. *Eur. J. Hum. Genet.* 11:3. 10.1038/sj.ejhg.5200946 12673279

[B35] De Marco P. Merello E. Consales A. Piatelli G. Cama A. Kibar Z. (2013). Genetic analysis of disheveled 2 and disheveled 3 in human neural tube defects. *J. Mol. Neurosci.* 49 582–588. 10.1007/s12031-012-9871-9 22892949 PMC3566388

[B36] De Marco P. Merello E. Rossi A. Piatelli G. Cama A. Kibar Z. (2011). FZD6 is a el gene for human neural tube defects. *Hum. Mutat.* 33 384–390. 10.1002/humu.21643 22045688 PMC3482927

[B37] Deak K. L. Boyles A. L. Etchevers H. C. Melvin E. C. Siegel D. G. Graham F. L. (2005a). SNPs in the neural cell adhesion molecule 1 gene (NCAM1) be associated with human neural tube defects. *Hum. Genet.* 117 133–142. 10.1007/s00439-005-1299-7 15883837 PMC3130962

[B38] Deak K. L. Dickerson M. E. Linney E. Enterline D. S. George T. M. Melvin E. C. (2005b). Analysis of ALDH1A2, CYP26A1, CYP26B1, CRABP1, and CRABP2 in human neural tube defects suggests a possible association with alleles in ALDH1A2. *Birt. Defects Res. A. Clin. Mol. Teratol.* 73 868–875. 10.1002/bdra.20183 16237707

[B39] Dong J. Horvath S. (2007). Understanding network concepts in modules. *BMC Syst. Biol.* 1:24. 10.1186/1752-0509-1-24 17547772 PMC3238286

[B40] Edilova M. I. Abdul-Sater A. A. Watts T. H. (2018). TRAF1 signaling in human health and disease. *Front. Immunol.* 9:2969. 10.3389/fimmu.2018.02969 30619326 PMC6305416

[B41] Enaw J. O. E. Zhu H. Yang W. Lu W. Shaw G. M. Lammer E. J. (2006). CHKA and PCYT1A gene polymorphisms, choline intake and spina bifida risk in a California population. *BMC Med.* 4:36. 10.1186/1741-7015-4-36 17184542 PMC1770928

[B42] Farooqui A. Tazyeen S. Mohd M. Ahmed A. Alam S. Ali M. D. (2018). Assessment of the key regulatory genes and their Interologs for Turner Syndrome employing network approach. *Sci. Rep.* 8:10091. 10.1038/s41598-018-28375-0 29973620 PMC6031616

[B43] Felder B. Stegmann K. Schultealbert A. Geller F. Strehl E. Ermert A. (2002). Evaluation of BMP4 and its specific inhibitor NOG as candidates in human neural tube defects (n.d.). *Eur. J. Hum. Genet. EJHG* 10 753–756. 10.1038/sj.ejhg.5200875 12404109

[B44] Findley T. Tenpenny J. C. O’Byrne M. I. R. Morrison A. C. Hixson J. E. Northrup H. (2017). Mutations in folate transporter genes and risk for human myelomeningocele. *Am. J. Med. Genet. A* 173 2973–2984. 28948692 10.1002/ajmg.a.38472 PMC5650522

[B45] Francesca L. C. Claudia R. Molinario C. Annamaria M. Chiara F. Natalia C. (2016). Variants in TNIP1, a regulator of the NF-kB pathway, found in two patients with neural tube defects. *Childs Nerv. Syst. CHNS Off. J. Int. Soc. Pediatr. Neurosurg.* 32 1061–1067. 10.1007/s00381-016-3087-1 27125519

[B46] Franke B. Vermeulen S. H. H. M. Steegers-Theunissen R. P. M. Marieke J. Scheffer H. Heijer M. D. (2009). An association study of 45 folate-related genes in spina bifida: involvement of cubilin (CUBN) and tRNA aspartic acid methyltransferase 1 (TRDMT1). *Br. Defects Res. Part A Clin. Mol. Teratol.* 85 216–226. 10.1002/bdra.20556 19161160

[B47] Fu Y. Wang L. Yi D. Jin L. Liu J. Zhang Y. (2015). Association between maternal single nucleotide polymorphisms in genes regulating glucose metabolism and risk for neural tube defects in offspring. *Br. Defects Res. A. Clin. Mol. Teratol.* 103 471–478. 10.1002/bdra.23332 25369983

[B48] Goll M. G. Kirpekar F. Maggert K. A. Yoder J. A. Hsieh C.-L. Zhang X. (2006). Methylation of tRNAAsp by the DNA methyltransferase homolog Dnmt2. *Science* 311 395–398. 10.1126/science.1120976 16424344

[B49] Gonzalez-Herrera L. Martín Cerda-Flores R. Luna-Rivero M. Canto-Herrera J. Pinto-Escalante D. Perez-Herrera N. (2010). Paraoxonase 1 polymorphisms and haplotypes and the risk for having offspring affected with spina bifida in Southeast Mexico. *Birt. Defects Res. A. Clin. Mol. Teratol.* 88 987–994. 10.1002/bdra.20727 21031563

[B50] Guan Z. Wang J. Guo J. Wang F. Wang X. Li G. (2014). The maternal ITPK1 gene polymorphism is associated with neural tube defects in a high-risk chinese population. *PLoS One* 9:e86145. 10.1371/journal.pone.0086145 24465924 PMC3896452

[B51] Guo J. Xie H. Wang J. Zhao H. Wang F. Liu C. (2013). The maternal folate hydrolase gene polymorphism is associated with neural tube defects in a high-risk Chinese population. *Genes Nutr.* 8 191–197. 10.1007/s12263-012-0309-3 22918695 PMC3575888

[B52] Hamosh A. Scott A. F. Amberger J. S. Bocchini C. A. McKusick V. A. (2005). Online Mendelian Inheritance in Man (OMIM), a knowledgebase of human genes and genetic disorders. *Nucleic Acids Res.* 33 D514–D517. 10.1093/nar/gki033 15608251 PMC539987

[B53] Hebert L. Hillman P. Baker C. Brown M. Ashley-Koch A. Hixson J. E. (2020). Burden of rare deleterious variants in WNT signaling genes among 511 myelomeningocele patients. *PLoS One* 15:e0239083. 10.1371/journal.pone.0239083 32970752 PMC7514064

[B54] Heyninck K. De Valck D. Berghe W. V. Van Criekinge W. Contreras R. Fiers W. (1999). The zinc finger protein A20 inhibits TNF-induced NF-κB-dependent gene expression by interfering with an RIP- or TRAF2-mediated transactivation signal and directly binds to a el NF-κB-inhibiting protein ABIN. *J. Cell Biol.* 145 1471–1482. 10.1083/jcb.145.7.1471 10385526 PMC2133159

[B55] Hicks J. A. Li L. Matsui M. Chu Y. Volkov O. Johnson K. C. (2017). Human GW182 paralogs are the central organizers for RNA-mediated control of transcription. *Cell Rep.* 20 1543–1552. 10.1016/j.celrep.2017.07.058 28813667 PMC5588873

[B56] Hol F. A. Geurds M. P. Chatkupt S. Shugart Y. Y. Balling R. Schrander-Stumpel C. T. (1996). PAX genes and human neural tube defects: an amino acid substitution in PAX1 in a patient with spina bifida. *J. Med. Genet.* 33 655–660. 10.1136/jmg.33.8.655 8863157 PMC1050699

[B57] Huang D. W. Sherman B. T. Lempicki R. A. (2009a). Bioinformatics enrichment tools: paths toward the comprehensive functional analysis of large gene lists. *Nucleic Acids Res.* 37 1–13. 10.1093/nar/gkn923 19033363 PMC2615629

[B58] Huang D. W. Sherman B. T. Lempicki R. A. (2009b). Systematic and integrative analysis of large gene lists using DAVID bioinformatics resources. *Nat. Protoc.* 4 44–57. 10.1038/nprot.2008.211 19131956

[B59] Idriss H. T. Naismith J. H. (2000). TNFα and the TNF receptor superfamily: structure-function relationship(s). *Microsc. Res. Tech.* 50 184–195. 10891884 10.1002/1097-0029(20000801)50:3<184::AID-JEMT2>3.0.CO;2-H

[B60] Jensen L. E. Etheredge A. J. Brown K. S. Mitchell L. E. Whitehead A. S. (2006a). Maternal genotype for the monocyte chemoattractant protein 1 A(-2518)G promoter polymorphism is associated with the risk of spina bifida in offspring. *Am. J. Med. Genet. A.* 140A 1114–1118. 10.1002/ajmg.a.31212 16596675

[B61] Jensen L. E. Hoess K. Mitchell L. E. Whitehead A. S. (2006b). Loss of function polymorphisms in NAT1 protect against spina bifida. *Hum. Genet.* 120 52–57. 10.1007/s00439-006-0181-6 16680433

[B62] Johnson K. J. Lee J. M. Ahsan K. Padda H. Feng Q. Partap S. (2017). Pediatric cancer risk in association with birth defects: a systematic review. *PLoS One* 12:e0181246. 10.1371/journal.pone.0181246 28749971 PMC5716403

[B63] Juriloff D. M. Harris M. J. (2012). A consideration of the evidence that genetic defects in planar cell polarity contribute to the etiology of human neural tube defects. *Birt. Defects Res. Part A Clin. Mol. Teratol.* 94 824–840. 10.1002/bdra.23079 23024041

[B64] Kase B. A. Northrup H. Au K. S. (2013). El single nucleotide polymorphisms in the superoxide dismutase 1 and 2 genes among children with myelomeningocele. *Am. J. Obstet. Gynecol.* 209 388–388. 10.1016/j.ajog.2013.06.004 23792044 PMC3786042

[B65] Kase B. A. Northrup H. Morrison A. C. Davidson C. M. Goiffon A. M. Fletcher J. M. (2012). Association of copper-zinc superoxide dismutase (SOD1) and manganese superoxide dismutase (SOD2) genes with nonsyndromic myelomeningocele. *Birt. Defects Res. A. Clin. Mol. Teratol.* 94 762–769. 10.1002/bdra.23065 22972774 PMC3506000

[B66] Kibar Z. Salem S. Bosoi C. M. Pauwels E. Marco P. D. Merello E. (2011). Contribution of VANGL2 mutations to isolated neural tube defects. *Clin. Genet.* 80 76–82. 20738329 10.1111/j.1399-0004.2010.01515.x PMC3000889

[B67] Kim S.-E. Lei Y. Hwang S.-H. Wlodarczyk B. J. Mukhopadhyay S. Shaw G. M. (2019). Dominant negative GPR161 rare variants are risk factors of human spina bifida. *Hum. Mol. Genet.* 28 200–208. 30256984 10.1093/hmg/ddy339 PMC6321953

[B68] King T. M. Au K.-S. Kirkpatrick T. J. Davidson C. Fletcher J. M. Townsend I. (2007). The impact of BRCA1 on spina bifida meningomyelocele lesions. *Ann. Hum. Genet.* 71 719–728. 10.1111/j.1469-1809.2007.00377.x 17640328

[B69] Klootwijk R. Groenen P. Schijvenaars M. Hol F. Hamel B. Straatman H. (2004). Genetic variants in ZIC1, ZIC2, and ZIC3 are not major risk factors for neural tube defects in humans. *Am. J. Med. Genet. A* 124A 40–47. 10.1002/ajmg.a.20402 14679585

[B70] Lavery W. J. Barski A. Wiley S. Schorry E. K. Lindsley A. W. (2020). KMT2C/D COMPASS complex-associated diseases [KCDCOM-ADs]: an emerging class of congenital regulopathies. *Clin. Epigenet.* 12:14582. 10.1186/s13148-019-0802-2 31924266 PMC6954584

[B71] Le P. De Marco P. Emond A. Spiegelman D. Dionne-Laporte A. Laurent S. (2017). Rare deleterious variants in GRHL3 are associated with human spina bifida. *Hum. Mutat.* 38 716–724. 10.1002/humu.23214 28276201

[B72] Le P. De Marco P. Traverso M. Merello E. Dionne-Laporte A. Spiegelman D. (2018). Whole exome sequencing identifies el predisposing genes in neural tube defects. *Mol. Genet. Genomic Med.* 7:467. 10.1002/mgg3.467 30415495 PMC6382446

[B73] Le P. Guyot M.-C. Tremblay É Dionne-Laporte A. Spiegelman D. Henrion É, et al. (2015). Loss-of-function de o mutations play an important role in severe human neural tube defects. *J. Med. Genet.* 52 493–497. 10.1136/jmedgenet-2015-103027 25805808

[B74] Lei Y. Fathe K. McCartney D. Zhu H. Yang W. Ross M. E. (2015). Rare LRP6 variants identified in spina bifida patients. *Hum. Mutat.* 36 342–349. 25546815 10.1002/humu.22750 PMC4361299

[B75] Lei Y. Kim S.-E. Chen Z. Cao X. Zhu H. Yang W. (2019). Variants identified in PTK7 associated with neural tube defects. *Mol. Genet. Genomic Med.* 7:e00584. 10.1002/mgg3.584 30689296 PMC6465732

[B76] Lei Y. Zhu H. Duhon C. Yang W. Ross M. E. Shaw G. M. (2013). Mutations in planar cell polarity gene SCRIB are associated with spina bifida. *PLoS One* 8:e69262. 10.1371/journal.pone.0069262 23922697 PMC3724847

[B77] Lei Y. Zhu H. Yang W. Ross M. E. Shaw G. M. Finnell R. H. (2014). Identification of el CELSR1 mutations in Spina Bifida. *PLoS One* 9:e92207. 10.1371/journal.pone.0092207 24632739 PMC3954890

[B78] Liu J. Xiong Q. Shi W. Shi X. Wang K. (2016). Evaluating the importance of nodes in complex networks. *Phys. Stat. Mech. ITS Appl.* 452 209–219. 10.1016/j.physa.2016.02.049

[B79] Lu W. Guzman A. R. Yang W. Chapa C. J. Shaw G. M. Greene R. M. (2010). Genes encoding critical transcriptional activators for murine neural tube development and human spina bifida: a case-control study. *BMC Med. Genet.* 11:141. 10.1186/1471-2350-11-141 20932315 PMC2964532

[B80] Lu X.-L. Wang L. Chang S.-Y. Shangguan S.-F. Wang Z. Wu L.-H. (2016). Sonic hedgehog signaling affected by promoter hypermethylation induces aberrant Gli2 expression in spina bifida. *Mol. Neurobiol.* 53 5413–5424. 10.1007/s12035-015-9447-0 26446020

[B81] Lu Z.-Y. Morales M. Khartulyari S. Mei M. Murphy K. M. Stanislawska-Sachadyn A. (2008). Genetic and biochemical determinants of serum concentrations of monocyte chemoattractant protein-1, a potential neural tube defect risk factor. *Birt. Defects Res. A. Clin. Mol. Teratol.* 82 736–741. 10.1002/bdra.20507 18937353 PMC3014088

[B82] Lupo P. J. Canfield M. A. Chapa C. Lu W. Agopian A. J. Mitchell L. E. (2012). Diabetes and obesity-related genes and the risk of neural tube defects in the national birth defects prevention study. *Am. J. Epidemiol.* 176 1101–1109. 10.1093/aje/kws190 23132673 PMC3571234

[B83] Malik M. Z. Alam M. J. Ishrat R. Agarwal S. M. Singh R. K. B. (2017). Control of apoptosis by SMAR1. *Mol. Biosyst.* 13 350–362. 10.1039/c6mb00525j 27934984

[B84] Malik M. Z. Chirom K. Ali S. Ishrat R. Somvanshi P. Singh R. K. B. (2019). Methodology of predicting el key regulators in ovarian cancer network: a network theoretical approach. *BMC Cancer* 19:1129. 10.1186/s12885-019-6309-6 31752757 PMC6869253

[B85] Mangangcha I. R. Malik M. D. Z. Küçük Ö Ali S. Singh R. K. B. (2019). Identification of key regulators in prostate cancer from gene expression datasets of patients. *Sci. Rep.* 9:16420. 10.1038/s41598-019-52896-x 31712650 PMC6848149

[B86] Mangangcha I. R. Malik M. Z. Kucuk O. Ali S. Singh R. K. B. (2020). Kinless hubs are potential target genes in prostate cancer network. *Genomics* 112 5227–5239. 10.1016/j.ygeno.2020.09.033 32976977

[B87] Martinez C. A. Northrup H. Lin J.-I. Morrison A. C. Fletcher J. M. Tyerman G. H. (2009). Genetic association study of putative functional single nucleotide polymorphisms of genes in folate metabolism and spina bifida. *Am. J. Obstet. Gynecol.* 201 394–394. 10.1016/j.ajog.2009.06.042 19683694 PMC2790326

[B88] Maslov S. Sneppen K. (2002). Specificity and stability in topology of protein networks. *Science* 296 910–913. 10.1126/science.1065103 11988575

[B89] Mason O. Verwoerd M. (2007). Graph theory and networks in biology. *IET Syst. Biol.* 1 89–119. 10.1049/iet-syb:20060038 17441552

[B90] Milazzo G. Mercatelli D. Di Muzio G. Triboli L. De Rosa P. Perini G. (2020). Histone Deacetylases (HDACs): evolution, specificity, role in transcriptional complexes, and pharmacological actionability. *Genes* 11:5. 10.3390/genes11050556 32429325 PMC7288346

[B91] Mitchell L. E. Adzick N. S. Melchionne J. Pasquariello P. S. Sutton L. N. Whitehead A. S. (2004). Spina bifida. *Lancet* 364 1885–1895. 10.1016/S0140-6736(04)17445-X 15555669

[B92] Mohd-Zin S. W. Marwan A. I. Abou Chaar M. K. Ahmad-Annuar A. Abdul-Aziz N. M. (2017). Spina Bifida: pathogenesis, mechanisms, and genes in mice and humans. *Scientifica* 2017:5364827. 10.1155/2017/5364827 28286691 PMC5327787

[B93] Nafis S. Kalaiarasan P. Brojen Singh R. K. Husain M. Bamezai R. N. K. (2015). Apoptosis regulatory protein-protein interaction demonstrates hierarchical scale-free fractal network. Brief. *Bioinformation* 16 675–699. 10.1093/bib/bbu036 25256288

[B94] Newman M. E. J. (2005). A measure of betweenness centrality based on random walks. *Soc. Netw.* 27 39–54. 10.1016/j.socnet.2004.11.009

[B95] Newman M. E. J. (2006). Finding community structure in networks using the eigenvectors of matrices. *Phys. Rev. E* 74:036104. 10.1103/PhysRevE.74.036104 17025705

[B96] Newman M. E. J. Girvan M. (2004). Finding and evaluating community structure in networks. *Phys. Rev. E* 69:026113. 10.1103/PhysRevE.69.026113 14995526

[B97] Nunes Amaral L. A. Guimera R. (2006). Lies, damned lies and statistics. *Nat. Phys.* 2 75–76. 10.1038/nphys228

[B98] O’Byrne M. R. Au K. S. Morrison A. C. Lin J.-I. Fletcher J. M. Ostermaier K. K. (2010). Association of folate receptor (folr1, folr2, folr3) and reduced folate carrier (slc19a1) genes with meningomyelocele. *Birt. Defects Res. A. Clin. Mol. Teratol.* 88 689–694. 10.1002/bdra.20706 20683905 PMC3046546

[B99] Olshan A. F. Shaw G. M. Millikan R. C. Laurent C. Finnell R. H. (2005). Polymorphisms in DNA repair genes as risk factors for spina bifida and orofacial clefts. *Am. J. Med. Genet. A* 135 268–273. 15887293 10.1002/ajmg.a.30713

[B100] Pangilinan F. Geiler K. Dolle J. Troendle J. Swanson D. A. Molloy A. M. (2008). Construction of a high resolution linkage disequilibrium map to evaluate common genetic variation in TP53 and neural tube defect risk in an irish population. *Am. J. Med. Genet. A* 146A 2617–2625. 10.1002/ajmg.a.32504 18798306 PMC2836760

[B101] Parle-McDermott A. Kirke P. N. Mills J. L. Molloy A. M. Cox C. O’Leary V. B. (2006). Confirmation of the R653Q polymorphism of the trifunctional C1-synthase enzyme as a maternal risk for neural tube defects in the Irish population. *Eur. J. Hum. Genet.* 14 768–772. 16552426 10.1038/sj.ejhg.5201603

[B102] Parle-McDermott A. Pangilinan F. O’Brien K. K. Mills J. L. Magee A. M. Troendle J. (2009). A common variant in MTHFD1L is associated with neural tube defects and mRNA splicing efficiency. *Hum. Mutat.* 30 1650–1656. 10.1002/humu.21109 19777576 PMC2787683

[B103] Pastor-Satorras R. Vázquez A. Vespignani A. (2001). Dynamical and correlation properties of the internet. *Phys. Rev. Lett.* 87:258701. 10.1103/PhysRevLett.87.258701 11736611

[B104] Raman K. (2010). Construction and analysis of protein-protein interaction networks. *Autom. Exp.* 2:2. 10.1186/1759-4499-2-2 20334628 PMC2834675

[B105] Rat E. Billaut I. L. Allorge D. Guidice J. M. L. Tellier M. Cauffiez C. (2006). Evidence for a functional genetic polymorphism of the human retinoic acid-metabolizing enzyme CYP26A1, an enzyme that be involved in spina bifida. *Birth Defects Res. Part A Clin. Mol. Teratol.* 76 491–498. 10.1002/bdra.20275 16933217

[B106] Ravasz E. Barabási A.-L. (2003). Hierarchical organization in complex networks. *Phys. Rev. E* 67:026112. 10.1103/PhysRevE.67.026112 12636753

[B107] Ravasz E. Somera A. L. Mongru D. A. Oltvai Z. N. Barabási A. L. (2002). Hierarchical organization of modularity in metabolic networks. *Science* 297 1551–1555. 10.1126/science.1073374 12202830

[B108] Re A. Molineris I. Caselle M. (2008). Graph theory analysis of genomics problems: community analysis of fragile sites correlations and of pseudogenes alignments. *Comput. Math. Appl.* 55 1034–1043. 10.1016/j.camwa.2006.12.100

[B109] Rebekah P. K. Tella S. Buragadda S. Tiruvatturu M. K. Akka J. (2017). Interaction between maternal and paternal SHMT1 C1420T predisposes to neural tube defects in the fetus: evidence from case-control and family-based triad approaches. *Birth Defects Res.* 109 1020–1029. 10.1002/bdr2.23623 28762673

[B110] Robertson J. C. Jorcyk C. L. Oxford J. T. (2018). DICER1 Syndrome: DICER1 mutations in rare cancers. *Cancers* 10:5. 10.3390/cancers10050143 29762508 PMC5977116

[B111] Robinson A. Partridge D. Malhas A. Castro S. C. P. D. Gustavsson P. Thompson D. N. (2013). Is LMNB1 a susceptibility gene for neural tube defects in humans? *Birt. Defects Res. A. Clin. Mol. Teratol.* 97 398–402. 10.1002/bdra.23141 23733478 PMC3738925

[B112] Rochtus A. Izzi B. Vangeel E. Louwette S. Wittevrongel C. Lambrechts D. (2015). DNA methylation analysis of Homeobox genes implicates HOXB7 hypomethylation as risk factor for neural tube defects. *Epigenetics* 10 92–101. 10.1080/15592294.2014.998531 25565354 PMC4622610

[B113] Rochtus A. Winand R. Laenen G. Vangeel E. Izzi B. Wittevrongel C. (2016). Methylome analysis for spina bifida shows SOX18 hypomethylation as a risk factor with evidence for a complex (epi)genetic interplay to affect neural tube development. *Clin. Epigenet.* 8:108. 10.1186/s13148-016-0272-8 27757173 PMC5064967

[B114] Safra N. Bassuk A. G. Ferguson P. J. Aguilar M. Coulson R. L. Thomas N. (2013). Genome-Wide association mapping in dogs enables identification of the homeobox gene, NKX2-8, as a genetic component of neural tube defects in humans. *PLoS Genet.* 9:1003646. 10.1371/journal.pgen.1003646 23874236 PMC3715436

[B115] Saraç M. Önalan E. Bakal Ü Tartar T. Aydın M. Orman A. (2016). Magnesium-permeable TRPM6 polymorphisms in patients with meningomyelocele. *Springerplus* 5:1703. 10.1186/s40064-016-3395-7 27757375 PMC5047867

[B116] Seo J. H. Zilber Y. Babayeva S. Liu J. Kyriakopoulos P. De Marco P. (2011). Mutations in the planar cell polarity gene, Fuzzy, are associated with neural tube defects in humans. *Hum. Mol. Genet.* 20 4324–4333. 10.1093/hmg/ddr359 21840926

[B117] Shangguan S. Wang L. Chang S. Lu X. Wang Z. Wu L. (2015). DNA methylation aberrations rather than polymorphisms of FZD3 gene increase the risk of spina bifida in a high-risk region for neural tube defects. *Birt. Defects Res. A. Clin. Mol. Teratol.* 103 37–44. 10.1002/bdra.23285 25131656

[B118] Shannon P. Markiel A. Ozier O. Baliga N. S. Wang J. T. Ramage D. (2003). Cytoscape: a software environment for integrated models of biomolecular interaction networks. *Genome Res.* 13 2498–2504. 10.1101/gr.1239303 14597658 PMC403769

[B119] Shaw G. M. Lu W. Zhu H. Yang W. Briggs F. B. S. Carmichael S. L. (2009). 118 SNPs of folate-related genes and risks of spina bifida and conotruncal heart defects. *BMC Med. Genet.* 10:49. 10.1186/1471-2350-10-49 19493349 PMC2700092

[B120] Shi Y. Ding Y. Lei Y.-P. Yang X.-Y. Xie G.-M. Wen J. (2012). Identification of el rare mutations of DACT1 in human neural tube defects. *Hum. Mutat.* 33 1450–1455. 10.1002/humu.22121 22610794

[B121] Spellicy C. J. Norris J. Bend R. Bupp C. Mester P. Reynolds T. (2018). Key apoptotic genes APAF1 and CASP9 implicated in recurrent folate-resistant neural tube defects. *Eur. J. Hum. Genet.* 26 420–427. 10.1038/s41431-017-0025-y 29358613 PMC5838979

[B122] Suazo J. Pardo R. Castillo S. Martin L. M. Rojas F. Santos J. L. (2013). Family-based association study between SLC2A1, HK1, and LEPR polymorphisms with myelomeningocele in Chile. *Reprod. Sci. Thousand Oaks Calif.* 20 1207–1214. 10.1177/1933719113477489 23427181 PMC3766345

[B123] Szklarczyk D. Morris J. H. Cook H. Kuhn M. Wyder S. Simoic M. (2017). The STRING database in 2017: quality-controlled protein-protein association networks, made broadly accessible. *Nucleic Acids Res.* 45 D362–D368. 10.1093/nar/gkw937 27924014 PMC5210637

[B124] Tang Y. Li M. Wang J. Pan Y. Wu F.-X. (2015). CytoNCA: a cytoscape plugin for centrality analysis and evaluation of protein interaction networks. *Biosystems* 127 67–72. 10.1016/j.biosystems.2014.11.005 25451770

[B125] Tian T. Cao X. Kim S.-E. Lin Y. L. Steele J. W. Cabrera R. M. (2020). FKBP8 variants are risk factors for spina bifida. *Hum. Mol. Genet.* 29 3132–3144. 10.1093/hmg/ddaa211 32969478 PMC7645715

[B126] Traag V. A. Krings G. Dooren P. V. (2013). Significant scales in community structure. *Sci. Rep.* 3 1–10. 10.1038/srep02930 24121597 PMC3796307

[B127] Traag V. A. Van Dooren P. Nesterov Y. (2011). Narrow scope for resolution-limit-free community detection. *Phys. Rev. E* 84:016114. 10.1103/PhysRevE.84.016114 21867264

[B128] van der Linden I. J. M. den Heijer M. Afman L. A. Gellekink H. Vermeulen S. H. H. M. Kluijtmans L. A. J. (2006). The methionine synthase reductase 66A>G polymorphism is a maternal risk factor for spina bifida. *J. Mol. Med. Berl. Ger.* 84 1047–1054. 10.1007/s00109-006-0093-x 17024475

[B129] Volcik K. A. Shaw G. M. Zhu H. Lammer E. J. Finnell R. H. (2003). Risk factors for neural tube defects: associations between uncoupling protein 2 polymorphisms and spina bifida. *Birt. Defects Res. A. Clin. Mol. Teratol.* 67 158–161. 10.1002/bdra.10019 12797456

[B130] Wang L. Lin S. Yi D. Huang Y. Wang C. Jin L. (2017). Apoptosis, expression of PAX3 and P53, and caspase signal in fetuses with neural tube defects. *Birth Defects Res.* 109 1596–1604. 10.1002/bdr2.1094 28786179

[B131] Wang Z. Shangguan S. Lu X. Chang S. Li R. Wu L. (2013a). Association of SMO polymorphisms and neural tube defects in the Chinese population from Shanxi Province. *Int. J. Clin. Exp. Med.* 6 960–966. 24260604 PMC3832335

[B132] Wang Z. Wang L. Shangguan S. Lu X. Chang S. Wang J. (2013b). Association between PTCH1 polymorphisms and risk of neural tube defects in a Chinese population. *Birt. Defects Res. A. Clin. Mol. Teratol.* 97 409–415. 10.1002/bdra.23152 23761049

[B133] Wen S. Lu W. Zhu H. Yang W. Shaw G. M. Lammer E. J. (2009). Genetic polymorphisms in the thioredoxin 2 (TXN2) gene and risk for spina bifida. *Am. J. Med. Genet. A* 149A 155–160. 10.1002/ajmg.a.32589 19165900 PMC2970524

[B134] Yang X. Y. Zhou X. Y. Wang Q. Q. Li H. Chen Y. Lei Y. P. (2013). Mutations in the COPII vesicle component gene SEC24B are associated with human neural tube defects. *Hum Mutat.* 34 1094–1101. 23592378 10.1002/humu.22338

[B135] Ye J. Tong Y. Lv J. Peng R. Chen S. Kuang L. (2020). Rare mutations in the autophagy-regulating gene AMBRA1 contribute to human neural tube defects. *Hum. Mutat.* 41 1383–1393. 10.1002/humu.24028 32333458

[B136] Zhang B. Li Z. Wang W. Guo J. Kang S. Liu S. (2018). NCOA3 Loss disrupts molecular signature of chondrocytes and promotes posttraumatic osteoarthritis progression. *Cell Physiol Biochem.* 49 2396–2413. 30261507 10.1159/000493839

[B137] Zhang H. Guo Y. Gu H. Wei X. Ma W. Liu D. (2019). TRIM4 is associated with neural tube defects based on genome-wide DNA methylation analysis. *Clin. Epigenet.* 11:17. 10.1186/s13148-018-0603-z 30709423 PMC6359777

[B138] Zhang X. Pei L. Li R. Zhang W. Yang H. Li Y. (2015). Spina bifida in fetus is associated with an altered pattern of DNA methylation in placenta. *J. Hum. Genet.* 60 605–611. 10.1038/jhg.2015.80 26178427

[B139] Zhang X.-F. Zhang R. Huang L. Wang P.-X. Zhang Y. Jiang D.-S. (2014). TRAF1 is a key mediator for hepatic ischemia/reperfusion injury. *Cell Death Dis.* 5:10. 10.1038/cddis.2014.411 25321474 PMC4649517

[B140] Zhu H. Enaw J. O. E. Ma C. Shaw G. M. Lammer E. J. Finnell R. H. (2007). Association between CFL1 gene polymorphisms and spina bifida risk in a California population. *BMC Med. Genet.* 8:12. 10.1186/1471-2350-8-12 17352815 PMC1831766

[B141] Zhu H. Wicker N. J. Volcik K. Zhang J. Shaw G. M. Lammer E. J. (2004). Promoter haplotype combinations for the human PDGFRA gene are associated with risk of neural tube defects. *Mol. Genet. Metab.* 81 127–132. 10.1016/j.ymgme.2003.11.003 14741194

[B142] Zhu H. Yang W. Lu W. Zhang J. Shaw G. M. Lammer E. J. (2006). A known functional polymorphism (Ile120Val) of the human PCMT1 gene and risk of spina bifida. *Mol. Genet. Metab.* 87 66–70. 10.1016/j.ymgme.2005.09.008 16256389 PMC2947858

[B143] Zou J. Wang F. Yang X. Wang H. Niswander L. Zhang T. (2020). Association between rare variants in specific functional pathways and human neural tube defects multiple subphenotypes. *Neural Dev.* 15:8. 10.1186/s13064-020-00145-7 32650820 PMC7353782

